# A Model Framework for Ion Channels with Selectivity Filters Based on Non-Equilibrium Thermodynamics

**DOI:** 10.3390/e27090981

**Published:** 2025-09-20

**Authors:** Christine Keller, Manuel Landstorfer, Jürgen Fuhrmann, Barbara Wagner

**Affiliations:** Weierstrass Institute for Applied Analysis and Stochastics (WIAS), Mohrenstr. 39, 10117 Berlin, Germany; manuel.landstorfer@wias-berlin.de (M.L.); juergen.fuhrmann@wias-berlin.de (J.F.); wagnerb@wias-berlin.de (B.W.)

**Keywords:** ion channel modeling, non-equilibrium thermodynamics, selective ion transport, Poisson–Nernst–Planck modeling, size-exclusion

## Abstract

A thermodynamically consistent model framework to describe ion transport in nanopores is presented. The continuum model unifies electro-diffusion and selective ion transport and extends the classical Poisson–Nernst–Planck (PNP) system for an idealized incompressible mixture by including finite ion size and solvation effects. Special emphasis is placed on the consistent modeling of the selectivity filter within the pore. It is treated as an embedded domain in which the constituents can change their chemical properties and mobility. Using this framework, we achieve good agreement with an experimentally observed current–voltage (IV) characteristic for an L-type selective calcium ion channel for a range of ion concentrations. In particular, we show that the model captures the experimentally observed anomalous mole fraction effect (AMFE). As a result, we find that calcium and sodium currents depend on the surface charge in the selectivity filter, the mobility of ions and the available space in the channel. Our results show that negative charges within the pore have a decisive influence on the selectivity of divalent over monovalent ions, supporting the view that AMFE can emerge from competition and binding effects in a multi-ion environment. Furthermore, the flexibility of the model allows its application in a wide range of channel types and environmental conditions, including both biological ion channels and synthetic nanopores, such as engineered membrane systems with selective ion transport.

## 1. Introduction

The intricate and fundamental processes governing cellular functions are orchestrated by a myriad of biological ion channels that regulate the movement of ions across cell membranes. Among these channels, calcium ion channels hold a prominent position due to their vital role in various physiological functions [1,2]. Understanding the complex mechanisms underlying calcium ion channels is of great importance, as they play a crucial role in cellular signaling, muscle contraction, neurotransmitter release, gene expression, and many other cellular processes [1,2,3,4]. Traditional experimental techniques, such as electrophysiology and X-ray crystallography, have significantly contributed to our understanding of ion channels [5,6]. However, studying ion channels using traditional experimental techniques is a complex and challenging task. As a result, the development of advanced modeling and simulation techniques has become essential in unraveling the mysteries of ion channels and providing deeper insights into their structure-function relationships and underlying mechanisms. This paper aims to explore the necessity of modeling and simulating biological ion channels, with a specific focus on ${\mathrm{Ca}}^{2+}$ ion channels, to bridge the gap between experimental observations and theoretical predictions. Computational modeling offers a powerful approach to complement experimental studies by providing in-depth insights into the behavior of ion channels at the molecular level. Molecular dynamics (MD) simulations, for example, can offer a very detailed description of the problem by resolving the structure of the ion channel protein at the atomic level. This allows the study of individual ion channels in a controlled environment, capturing their conformational dynamics and interactions with ions and other molecules [7,8]. MD simulations could, for example, contribute to a better understanding of the selectivity mechanisms within ion channels, especially in KcsA channels [9,10,11].

Understanding the molecular mechanisms of ${\mathrm{Ca}}^{2+}$ ion channels is not merely an academic pursuit; it holds significant implications for human health and disease. Dysregulation of these channels has been linked to a wide range of pathologies, including cardiac arrhythmias, neurodegenerative disorders, and cancer [1,2,12,13]. By gaining a comprehensive understanding of ${\mathrm{Ca}}^{2+}$ ion channels through modeling and simulation, researchers can identify potential therapeutic targets for drug development and design more efficient and targeted interventions.

However, the molecular description is often computationally intensive, which results, for instance, in a limitation of the simulated time span. This, on the other hand, can lead to an incomplete depiction of experimental observations, which often span several seconds [14,15]. A formulation of the problem on a coarse-grained macroscopic level can address this issue. Most such continuum models are based on Poisson–Nernst–Planck (PNP) theory, which has been successfully used to simulate semiconductor devices [2,16,17] and can be derived by averaging a Poisson-Langevin model [18]. Although not every atomistic detail is resolved, the PNP theory can make predictions about current–voltage (IV) relations and capture high variations in ion concentration, e.g., ${\mathrm{Ca}}^{2+}$ concentrations that range from $10^{-8}$ to $10^{-6}$ M. However, several challenges persist, such as accurately representing ion–ion and ion–protein interactions, capturing solvent effects, and developing reliable force fields for membrane proteins. Great efforts have been made to overcome these problems, and the Poisson–Nernst–Planck–Bikerman (PNPB) theory has been developed [19]. Modified chemical potential functions with steric effects were formulated to account for size-exclusion effects and to include water molecules. For this purpose, expressions for entropy are often derived based on thermodynamic principles, such as density functional theory (DFT) [20,21] or mean spherical approximation (MSA) [19,22,23]. An alternative approach is to derive a continuum formulation directly from a stochastic formulation such as hopping models [24]. As computational techniques continue to evolve, the integration of multi-scale simulations and machine learning approaches holds the promise of unraveling even more complex behaviors of ${\mathrm{Ca}}^{2+}$ ion channels. For example, MD simulations can be used to find stable ion configurations and calculate the channel geometry, which is then used to solve the continuum model [25,26]. In this work, we derive a continuum framework for ion channels in a liquid electrolyte environment based on non-equilibrium thermodynamics [27]. It provides a consistent coupling of diffusion and mechanics so that the conservation of mass is fulfilled for the whole system. It couples the momentum balance to the Nernst-Planck system, which is used to determine the evolution of the solvent [28,29]. The material modeling is based on the derivation of the free energy density, which allows different system properties within different phases, such as the intracellular or extracellular, to be taken into account.

Our work puts a special emphasis on a consistent modeling of the selectivity filter within the ion channel, since it is the crucial structural element that governs the highly selective movement of specific ions across the cell membrane [6,11]. Only certain ions are allowed to pass through the cell membrane in order to maintain ion homeostasis and to regulate various cellular processes. A key feature of the selectivity filter is its geometrically narrow pore region lined with specific amino acids or residues that form a highly structured environment. The size and shape of the pore dictate which ions can pass through, as it must accommodate the size and coordination requirements of the preferred ion. The selectivity filter is designed to coordinate and stabilize specific ions through electrostatic interactions and coordination bonds. These interactions help to overcome the energetic barrier that ions encounter when moving through the hydrophobic membrane. Each type of ion channel exhibits distinct ion selectivity, favoring the passage of certain ions over others. For instance, ${\mathrm{Ca}}^{2+}$ ion channels will preferentially allow the passage of calcium ions, while $K^{+}$ ion channels will primarily facilitate the movement of potassium ions. Notably, the amino acid residues that form the selectivity filter are often highly conserved among members of the same ion channel family, highlighting their crucial functional role and evolutionary significance. In some ion channels, the selectivity filter can also participate in the gating process, regulating the opening and closing of the channel in response to various stimuli, such as changes in membrane voltage or ligand binding. Overall, the selectivity filter in biological ion channels is a finely tuned structure that ensures the precise regulation of ion flux, enabling cells to maintain electrical and chemical gradients essential for cellular function and signaling. We include the selectivity filter as a separate phase to ensure the consistency of the whole model. The narrowest part of the filter is about the same size as the desolvated ions. Before the solvated ions enter the selectivity filter, they strip off the hydration shell. This phenomenon is accounted for by surface reactions at the interfaces between the outer (inner) region and the selectivity filter region. The electrostatic forces are integrated by a backbone charge and a surface charge.

In Section 2, the model framework is presented. The considered domain consists of different phases such as the intra- and extracellular regions and the selectivity filter (Section 2.1). Different species with different properties can be assigned to each phase (Section 2.2). In Section 2.3, the general system of equations is discussed with a detailed description of the chemical potential functions in Section 2.4. We consider different classes of boundary conditions (Section 2.6), and due to some equilibrium assumptions, the model can be reduced (Section 2.7), which is summarized in Section 2.8. In Section 3, we present our results on the impact and function of the selectivity filter and discuss comparisons to experimental data.

## 2. Methods

**Figure 1:**
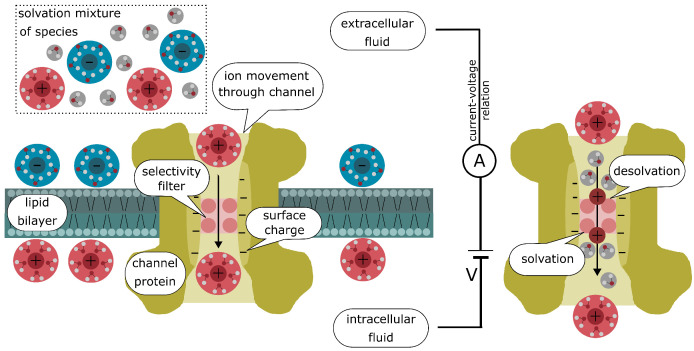
Illustration of an ion channel embedded in a lipid bilayer (cyan). The intracellular and extracellular regions contain a solvation mixture of species (cations (red), anions (blue) and solvent moelcules (gray)). The selectivity filter (pink) is located within the ion channel. The channel itself is formed by a pore-forming protein (yellow). On the right is an illustration of the desolvation and solvation reactions, as the ions usually pass through the filter without the solvation shell.

Our model takes into account various important aspects of the ion channel and its microenvironment, such as the solvation mixture of the species in the different regions as well as the surface charges, e.g., of the protein. An illustration of the most important features can be found in Figure 1. The ions in the intra- and extracellular regions are in a solvated state and are surrounded by hydration shells, which significantly influence their physical properties. This solvation increases both the effective volume and the mass of the ions and is therefore a decisive factor in the formulation of the model. The selectivity filter is located within the channel pore, where desolvation reactions are expected at the interfaces. When ions pass through this narrow region, they must shed part of their hydration shells, resulting in a locally dehydrated state. Since the removal of the solvation shell requires overcoming an energy barrier, ions must expend a desolvation cost to enter the filter. This energy barrier is reflected by the interface reactions. The structure and chemistry of the selectivity filter are tuned to compensate for this cost in a highly ion-specific manner, stabilizing certain ions more effectively than others. This balance between desolvation cost and filter coordination energy is a key mechanism by which some ion channels achieve their remarkable selectivity. In addition, desolvation changes the effective size, mass, and mobility and leads to different chemical properties of ions, which must be taken into account in the model formulation. Once the ions exit the selectivity filter, they rehydrate and regain their original solvated form, restoring their bulk transport characteristics.

Understanding these local variations in ion properties is essential for accurate modeling of ion transport through the channel, especially in the selectivity filter region where most of the functional differentiation takes place. This is crucial for the interpretation of experimentally measured IV relations and for gaining insight into physiologically relevant behaviors such as ion selectivity and conductivity. Finally, we can calculate the current *I* flowing from one bath to another as follows:

(1) $$\begin{array}{c} I=F\sum_{\alpha =0}^{N}\int_{S^{0}}z_{\alpha }J_{\alpha }\;\cdot \;n\;dS, \end{array}$$

where $S^{0}\subset S^{j}$ is a subset of the Dirichlet boundary, *F* is the Faraday constant, $z_{\alpha }$ is the charge number, and $J_{\alpha }$ is the flux. This becomes particularly important, for example, when examining the IV relationships for different membrane potentials *E*

(2) $$\begin{array}{c} E=\varphi^{\mathrm{in}}-\varphi^{\mathrm{out}}, \end{array}$$

with $\varphi^{\mathrm{out}}$ and $\varphi^{\mathrm{in}}$ being the potentials at the top and the bottom Dirichlet boundary condition, respectively.

### 2.1. Domains

**Figure 2:**
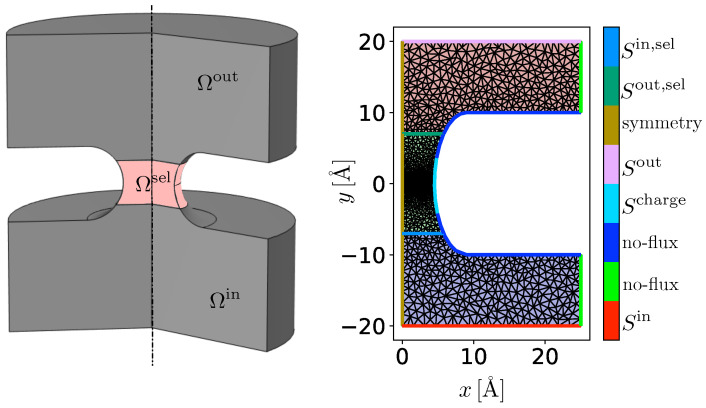
**Left**: Illustration of the rotationally symmetric domain. The top and the bottom bath (gray areas) are separated by an impermeable membrane (cut out) that contains a single pore. Inside the pore is the selectivity filter embedded as an additional domain (pink area). The full domain Ω is split into the outer bath $\Omega^{\mathrm{out}}$, the inner bath $\Omega^{\mathrm{in}}$, and the selectivity filter domain $\Omega^{\mathrm{sel}}$. **Right**: Simulation domain illustrating the shared interfaces between the different domains.

At the top of the considered domain, we have the outer bath $\Omega^{\mathrm{out}}$ and at the bottom the inner bath $\Omega^{\mathrm{in}}$, both of which are separated by an impermeable lipid bilayer. Inside this membrane there is a single pore, the ion channel, which allows the exchange of particles between the two baths. Within the pore there is an additional domain that controls actual physicochemical processes occurring inside this ion channel, termed selectivity filter $\Omega^{\mathrm{sel}}$. We consider a very idealized simple cylindrical, rotationally symmetric domain $\Omega \in R^{3}$ that is separated into different phases $\Omega^{j}$, $j\in J_{\Omega }:=\left\{\mathrm{out},\mathrm{in},\mathrm{sel}\right\}$. The protein and the lipid bilayer are treated as fixed domain boundaries. An illustration is given in Figure 2.

The domains $\Omega^{j}$, $j\in J_{\Omega }$ share several common interfaces $S^{j,k},j,k\in J_{\Omega },j\neq k$, e.g., the interface $S^{\mathrm{out},\mathrm{sel}}$ between the outer domain and the selectivity filter. The evaluation of some quantity, for example, the flux $J_{\alpha }$ at the *j*-side of an interface $S^{j,k}$ will, in general, be written as $J_{\alpha }{|}_{j}^{j,k}$, for instance, $J_{\alpha }{|}_{\mathrm{out}}^{\mathrm{out},\mathrm{sel}}$. If the interface is an actual boundary of a domain to the exterior, i.e., $S^{\mathrm{out}}$, then $J_{\alpha }{|}^{\mathrm{out}}$ is the evaluation of $J_{\alpha }$ at $S^{\mathrm{out}}$ approaching always from within the corresponding domain. In order to compactify the typeface, we will also use the typeface $J_{\alpha }{{|}_{j}^{j,k}=J_{\alpha }|}_{+}^{j,k}$ and $J_{\alpha }{{|}_{k}^{j,k}=J_{\alpha }|}_{-}^{j,k}$. Hence we define the jump brackets ${\left[\;\left[J_{\alpha }\right]\;\right]}^{j,k}:={J_{\alpha }}_{+}^{j,k}-{J_{\alpha }}_{-}^{j,k},j,k\in J_{\Omega }$. If no index ^*j,k*^ is given for the interface, then it is assumed to be implicitly clear from the context.

### 2.2. Species

**Figure 3:**
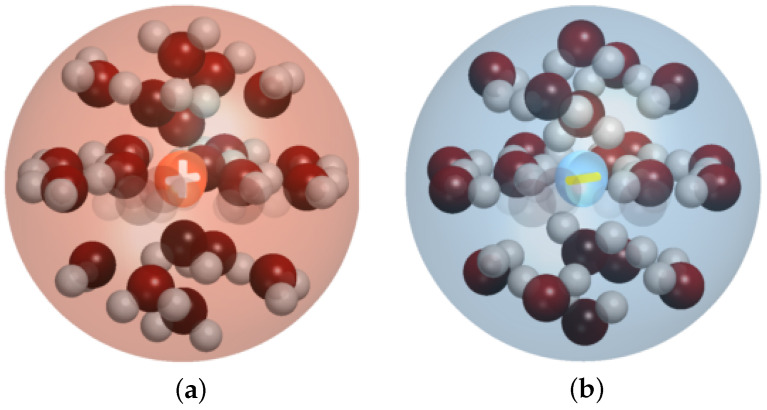
Illustration of (**a**) a monovalent cation (orange sphere with “+”) surrounded by water molecules (red spheres with two attached white spheres), forming a hydration shell (light-red) and (**b**) a monovalent anion (blue sphere with“–”) surrounded by water molecules, forming a hydration shell (light-blue).

In each region $\Omega^{j},j\in J_{\Omega }$, we consider a mixture of anions, cations, solvent molecules, and additional species. Each mixture contains $N^{j},j\in J_{\Omega },$ different constituents $A_{\alpha }^{j}$ with $\alpha \in I^{j}\subset N_{0}^{+}$. Quite frequently, the solvent is denoted by $A_{0}$, if present. The constituents have molar masses $m_{\alpha }^{j}$, molar volumes $v_{\alpha }^{j}$, and carry a charge $z_{\alpha }^{j}e_{0}$, where $e_{0}$ is the elementary charge. We emphasize that the ionic species are subject to solvation effects, as illustrated in Figure 3, whereby $m_{\alpha }^{j}$ and $v_{\alpha }^{j}$ denote the mass and volume of the solvated ions, respectively [29,30]. Hence, the molar mass is written as $m_{\alpha }^{j}={\tilde{m}}_{\alpha }+\kappa_{\alpha }^{j}m_{0}$ since mass is conserved upon solvation, where ${\tilde{m}}_{\alpha }$ is the mass of the central ion, $\kappa_{\alpha }^{j}$ the number of solvent molecules bound to the ion, and $m_{0}$ is the mass of the solvent molecule. For the partial molar volume of the solvated ions, a similar relation expectably holds, but the volume is not necessarily conserved upon solvation, whereby we have $v_{\alpha }^{j}\approx {\tilde{v}}_{\alpha }+\kappa_{\alpha }^{j}v_{0}$ with the molar volume ${\tilde{v}}_{\alpha }$ of the central ion and $v_{0}$ of the solvent. A convenient, useful, and meaningful approximation is, for example, $m_{\alpha }^{j}/m_{0}=v_{\alpha }^{j}/v_{0}$ [29]. The molar density for species $A_{\alpha }^{j},\alpha \in I^{j}$ is denoted as $n_{\alpha }^{j}\left(x,t\right)$ for $x\in \Omega^{j}$ with $j\in J_{\Omega }$. Further, the mass density $\rho^{j}\left(x,t\right)$ and the charge density $q^{j}\left(x,t\right)$ are given by

$$\rho^{j}=\sum_{\alpha \in I^{j}}m_{\alpha }^{j}n_{\alpha }^{j}\;\mathrm{and}\;q^{j}=F\sum_{\alpha \in I^{j}}z_{\alpha }^{j}n_{\alpha }^{j}.$$

### 2.3. Balance Equations

The continuum model is based on a modified Nernst–Planck system that accounts for ion-specific effects such as finite size and solvation and is coupled with the Poisson equation to describe electrostatic interactions. To account for fluid motion and pressure-induced effects within the channel, the system is further coupled with the Stokes equations, resulting in a thermodynamically consistent framework for ion transport in confined charged geometries. The ion flow through the channel is determined by the gradient of the chemical potential and the gradient of the electrostatic potential.

In the following, we assume that the process is isothermal, i.e., the temperature *T* is constant. The evolution of the molar densities $n_{\alpha }^{j}\left(x,t\right)$ for $\alpha \in I^{j},j\in J_{\Omega }$, the electrostatic potential φ(x,t) and the barycentric velocity v(x,t) for $x\in \Omega^{j},j\in J_{\Omega }$, are described by

(3) $$\begin{array}{cc} \partial_{t}n_{\alpha }^{j}+\nabla \;\cdot \;\left(n_{\alpha }^{j}v+J_{\alpha }^{j}\right) & =0\;\forall \alpha \in I^{j}, \end{array}$$

(4) $$\begin{array}{cc} -\nabla \;\cdot \;\left[\varepsilon_{0}\left(1+\chi^{j}\right)\nabla \varphi \right] & =q^{j}, \end{array}$$

(5) $$\begin{array}{cc} \nabla \;\cdot \;v & =0, \end{array}$$

(6) $$\begin{array}{cc} -\nu \Delta v+\nabla p+F\sum_{\alpha \in I^{j}}z_{\alpha }^{j}n_{\alpha }^{j}\nabla \varphi & =0. \end{array}$$

Note that diffusional fluxes $J_{\alpha }^{j}$ in Equation (3) are subject to the constraint

(7) $$\begin{array}{c} \sum_{\alpha \in I^{j}}m_{\alpha }^{j}J_{\alpha }^{j}=0. \end{array}$$

If the species $A_{0}$ is mobile, this condition can be exploited to show, based on non-equilibrium thermodynamics [27], that the flow is driven by the diffusive chemical potential ${\hat{\mu }}_{\alpha }^{j}=\mu_{\alpha }^{j}-\frac{m_{\alpha }^{j}}{m_{0}}\mu_{0}^{j}$, as well as by the electrostatic potential φ, and is given by

(8) $$\begin{array}{cc} J_{\alpha }^{j} & =-\sum_{\beta \in I^{j}}M_{\alpha \beta }^{j}\left(\nabla {\hat{\mu }}_{\beta }^{j}+Fz_{\beta }^{j}\nabla \varphi \right),\;\forall \alpha \in I^{j}, \end{array}$$

where the mobility matrix $M_{\alpha ,\beta }^{j}$ must be positive definite. The total mass flux of a species $A_{\alpha }^{j}$ is denoted by $j_{\alpha }^{j}:=n_{\alpha }^{j}v+J_{\alpha }^{j}$. In the Poisson equation (Equation (4)), we denote with $\varepsilon_{0}$ the vacuum permittivity, with the relative permittivity $\varepsilon_{r}^{j}=1+\chi^{j}$ and with $\chi^{j}$ the dielectric susceptibility. Note that we assume that $\chi^{j}$ is piecewise constant throughout this work; however, it might also dependent on the (local) species densities as well as on the electric field [31]. The Stokes equations (Equations (5) and (6)) consist of the continuity equation and the momentum balance equation, with the material pressure *p* and the electric field E=−∇φ.

Besides the Poisson equation (Equation (4)), in some situations it is convenient to consider also the charge balance equation

(9) $$\begin{array}{c} \partial_{t}q^{j}+\nabla \;\cdot \;\left(q^{j}v+J_{q}^{j}\right)=0, \end{array}$$

where $J_{q}^{j}=F\sum_{\alpha \in I^{j}}z_{\alpha }^{j}J_{\alpha }^{j}$ is the (diffusional) electric charge and $j_{q}^{j}:=q^{j}v+J_{q}^{j}$ the total charge of the system.

### 2.4. Chemical Potential Functions

For each constituent $A_{\alpha }^{j}$, we have a chemical potential $\mu_{\alpha }^{j}$, which is determined from the free energy density $\rho \psi^{j}$ of the mixture in the respective phase $\Omega^{j},j\in J_{\Omega }$, i.e.,

(10) $$\begin{array}{c} \mu_{\alpha }^{j}:=\frac{\partial (\rho \psi^{j})}{\partial n_{\alpha }^{j}},\;\forall \alpha \in I^{j}. \end{array}$$

Note that the above syntax with superscript ^*j*^ allows us to distinguish for a specific ion, e.g., ${\mathrm{Ca}}^{2+}$, its actual state in the various phases of our system Ω. For example, the constituent ${\mathrm{Ca}}^{2+}$ is solvated in the electrolytic domain $\Omega^{\mathrm{in}}$ but desolvated in $\Omega^{\mathrm{sel}}$. To account for this, we have to denote ${\mathrm{Ca}}^{2+}$ in $\Omega^{\mathrm{in}}$ as ${\mathrm{Ca}}^{2+,\mathrm{in}}$ and ${\mathrm{Ca}}^{2+}$ in $\Omega^{\mathrm{sel}}$ as ${\mathrm{Ca}}^{2+,\mathrm{sel}}$, and consequently all the corresponding material functions, such as $n_{\alpha }$ or $\mu_{\alpha }$. If, for a given species, the assignment is unique, we drop the index ^*j*^.

#### 2.4.1. Liquid Electrolyte

Within the bulk regions $\Omega^{j},j=\left\{\mathrm{out},\mathrm{in}\right\}$ we consider a liquid electrolyte mixture of several charged and uncharged constituents. The charged ions are subject to the solvation effect, which is of major importance for the material models [29,30]. As mentioned in Section 2.2, solvation has an effect on the total mass and the total volume of the solvated ion, which must be taken into account. Thus, the chemical potential function $\mu_{\alpha }^{j}$ accounts entropically for solvation effects. In the incompressible limit we derive the following chemical potential function for a liquid electrolyte:

(11) $$\begin{array}{c} \mu_{\alpha }^{j}=g_{\alpha }^{j}+RT\;\ln\left(y_{\alpha }^{j}\right)+v_{\alpha }^{j}\;p, \end{array}$$

where $g_{\alpha }^{j}$ is a reference potential that accounts energetically for the solvation effect, $y_{\alpha }^{j}\;=\;n_{\alpha }^{j}/n^{j}$ is the mole fraction, $n^{j}$ is the total density, $v_{\alpha }^{j}$ denotes the partial molar volume of the (solvated) constituent in the phase $\Omega^{j}$, *p* the material pressure, *R* the gas constant, and *T* the temperature. A detailed derivation of the chemical potential is given in the Appendix A and in [28,32]. The incompressible limit entails further the incompressibility constraint.

(12) $$\begin{array}{c} \sum_{\alpha \in I^{j}}v_{\alpha }^{j}n_{\alpha }^{j}=1, \end{array}$$

which allows us to express

(13) $$\begin{array}{c} n^{j}=\sum_{\alpha \in I^{j}}n_{\alpha }^{j}=\frac{1}{v_{0}}+\sum_{\alpha \in I^{j}\setminus 0}\left(1-\frac{v_{\alpha }^{j}}{v_{0}}\right)n_{\alpha }^{j}=n_{0}^{\mathrm{ref}}+\sum_{\alpha \in I^{j}\setminus 0}\left(1-n_{0}^{\mathrm{ref}}v_{\alpha }^{j}\right)n_{\alpha }^{j}, \end{array}$$

and

(14) $$\begin{array}{c} y_{\alpha }^{j}=\frac{n_{\alpha }^{j}}{n_{0}^{\mathrm{ref}}+\sum_{\beta \in I^{j}\setminus 0}\left(1-n_{0}^{\mathrm{ref}}v_{\beta }^{j}\right)n_{\beta }^{j}}, \end{array}$$

where $n_{0}^{\mathrm{ref}}=\mathrm{const}.$ is the reference molar density of the pure solvent, e.g., $n_{0}^{\mathrm{ref}}=55.5$ M. The diffusional flux in Equation (8) becomes

(15) $$\begin{array}{c} J_{\alpha }^{j}=-\sum_{\beta \in I^{j}}M_{\alpha \beta }^{j}\left(RT\;\nabla (\ln\left(y_{\beta }^{j}\right)-\frac{m_{\beta }^{j}}{m_{0}}\ln\left(y_{0}\right))+(v_{\beta }^{j}-\frac{m_{\beta }^{j}}{m_{0}}v_{0})\nabla p+Fz_{\beta }^{j}\nabla \varphi \right) \end{array}$$

#### 2.4.2. Polymeric Electrolyte

This work focuses on the mathematically consistent modeling of the selectivity filter. Our approach is to include it as an additional embedded domain and treat it as a polymeric or solid electrolyte. The assumption is that the filter region is formed by immobile scaffold-forming species that mix and interact with the channel passing ions. The underlying idea is that within the selectivity filter region multiple binding sites exist and that the permeating ions hop from one site to the other [33]. We account for binding sites and the hopping of ions by assuming that the immobile species form lattice sites on which channel-passing species can move, i.e., ions can thus only move from one lattice site to another. Since in some ion channels such as voltage-gated calcium (${\mathrm{Ca}}_{v}$) channels the binding sites are formed by flexible side chains of the protein rather than a fixed scaffold, we will also introduce an additional free-moving species that is confined within the selectivity filter region. For example, in ${\mathrm{Ca}}_{v}$1.2 channels the oxygen ions of carboxylate groups from the amino acid residues of the EEEE locus form three binding sites for the passing calcium ions [34]. Those site chains coordinate the movement of ions through the channel and replace the hydration shell. Since the oxygen ions are tied to a fixed backbone, we will consider a mixing between the scaffold-forming species, the channel-passing ions, and the oxygen ions. Thus, oxygen ions can diffuse on the lattice sites but are confined within the selectivity filter region [35]. This idea is incorporated in the model within the free energy density by the mixing entropy. The chemical potential function $\mu_{\alpha }^{\mathrm{sel}}$ entropically accounts for desolvated ions, while the reference potential $g_{\alpha }^{\mathrm{sel}}$ energetically accounts for it.

We will denote the immobile scaffold-forming species as $A_{\alpha }^{\mathrm{scaf}},\alpha \in I^{\mathrm{scaf}}$. This species forms the lattice sites on which the mobile species $A_{\alpha }^{\mathrm{pass}},\;\alpha \in I^{\mathrm{pass}}$ may diffuse. The confined but diffusing species is denoted with $A_{\alpha }^{\mathrm{conf}},\;\alpha \in I^{\mathrm{conf}}$. We consider thus a mixture of particles on a lattice with $A_{\alpha }^{\mathrm{scaf}}\cup A_{\alpha }^{\mathrm{pass}}\cup A_{\alpha }^{\mathrm{conf}}=A_{\alpha }^{\mathrm{sel}}$ and $I^{\mathrm{scaf}}\cup I^{\mathrm{pass}}\cup I^{\mathrm{conf}}=I^{\mathrm{sel}}$. In the incompressible limit, we obtain the chemical potential functions for $\alpha \in I^{\mathrm{scaf}}$.

(16a) $$\begin{array}{cc} \mu_{\alpha }^{\mathrm{scaf}} & =\frac{\partial \rho \psi^{\mathrm{sel}}}{\partial n_{\alpha }}\\ \; & =g_{\alpha }^{\mathrm{sel}}+RT\;\omega_{\alpha }^{\mathrm{scaf}}\ln\left(y_{V}\right)+v_{\alpha }^{\mathrm{sel}}\;p+RT\;\omega_{\alpha }^{\mathrm{scaf}}\left(1-y_{\nu }-\sum_{\beta \in I^{\mathrm{pass}}\cup I^{\mathrm{conf}}}y_{\beta }\right), \end{array}$$

for $\alpha \in I^{\mathrm{conf}}$

(16b) $$\begin{array}{cc} \mu_{\alpha }^{\mathrm{conf}} & =\frac{\partial \rho \psi^{\mathrm{sel}}}{\partial n_{\alpha }}\\ \; & =g_{\alpha }^{\mathrm{sel}}+RT\;\ln\left(y_{\alpha }\right)-RT\;\omega_{\alpha }^{\mathrm{conf}}\ln\left(y_{V}\right)\\ \; & \;+RT\;\left(1-\omega_{\alpha }^{\mathrm{conf}}\right)\left(1-y_{\alpha }-y_{\nu }-\sum_{\beta \in I^{\mathrm{pass}}}y_{\beta }\right), \end{array}$$

and for $\alpha \in I^{\mathrm{pass}}$

(16c) $$\begin{array}{cc} \mu_{\alpha }^{\mathrm{pass}} & =\frac{\partial \rho \psi^{\mathrm{sel}}}{\partial n_{\alpha }}\\ \; & =g_{\alpha }^{\mathrm{sel}}+RT\;\ln\left(y_{\alpha }\right)-RT\;\omega_{\alpha }^{\mathrm{pass}}\ln\left(y_{V}\right)\\ \; & \;+RT\;\left(1-\omega_{\alpha }^{\mathrm{pass}}\right)\left(1-y_{\alpha }-y_{\nu }-\sum_{\beta \in I^{\mathrm{conf}}}y_{\beta }\right), \end{array}$$

where *p* denotes again the material pressure and with

(17) $$\begin{array}{cccc} \; & \tilde{n}=\sum_{\alpha \in I^{\mathrm{pass}}}n_{\alpha }+\sum_{\alpha \in I^{\mathrm{conf}}}n_{\alpha }+n_{V},\; & \; & \mathrm{total}\;\mathrm{number}\;\mathrm{of}\;\mathrm{particles}, \end{array}$$

(18) $$\begin{array}{cccc} \; & n_{\ell }=\sum_{\alpha \in I^{\mathrm{scaf}}}\omega_{\alpha }^{\mathrm{scaf}}n_{\alpha },\; & \; & \mathrm{the}\;\mathrm{number}\;\mathrm{of}\;\mathrm{lattice}\;\mathrm{sites},\; \end{array}$$

(19) $$\begin{array}{cccc} \; & n_{V}=n_{\ell }-\sum_{\alpha \in I^{\mathrm{pass}}}\omega_{\alpha }^{\mathrm{pass}}n_{\alpha }-\sum_{\alpha \in I^{\mathrm{conf}}}\omega_{\alpha }^{\mathrm{conf}}n_{\alpha },\; & \; & \mathrm{the}\;\mathrm{number}\;\mathrm{of}\;\mathrm{vacancies}, \end{array}$$

(20) $$\begin{array}{cccc} \; & y_{\alpha }:=\frac{n_{\alpha }}{\tilde{n}},\;\alpha \in \left\{I^{\mathrm{pass}}\cup I^{\mathrm{conf}},V\right\},\; & \; & \mathrm{the}\;\mathrm{lattice}\;\mathrm{fraction}, \end{array}$$

where V is the number of available lattice sites, i.e., vacancies, $\omega_{\alpha }^{\mathrm{scaf}},\alpha \in I^{\mathrm{scaf}}$ is the number of lattice sites each constituent $A_{\alpha }^{\mathrm{scaf}}$ delivers, and $\omega_{\alpha }^{\mathrm{conf}},\alpha \in I^{\mathrm{conf}}$ and $\omega_{\alpha }^{\mathrm{pass}},\alpha \in I^{\mathrm{pass}}$ are the number of lattice sites each constituent $A_{\alpha }^{\mathrm{conf}}$ and $A_{\alpha }^{\mathrm{pass}}$ requires on the lattice, respectively.

For the sake of simplicity we consider in the following that the lattice is built by a single species $A^{\mathrm{scaf}}=A_{0}^{\mathrm{sel}}$ whereby $n_{\ell }=\omega^{\mathrm{scaf}}n_{0}^{\mathrm{sel}}$ and that there is only one confined species $A^{\mathrm{conf}}=A_{N+1}^{\mathrm{sel}}$ that requires only a single lattice site $\omega^{\mathrm{conf}}=1$. Further, we assume that all species that are allowed to pass require only a single site, which employs $\omega_{\alpha }^{\mathrm{pass}}=1$, $\alpha \;\in \;I^{\mathrm{pass}}$. The lattice fraction of the vacancies can be rewritten as $y_{V}\;=\;1\;-\;\sum_{\beta \in I^{\mathrm{pass}}\cup I^{\mathrm{conf}}}y_{\beta }$, where $\tilde{n}=n_{\ell }=$ const. Further, we assume that $A_{0}^{\mathrm{sel}}$ is in equilibrium whereby $\nabla \mu_{0}=0$. Hence, we have for the diffusional flux ${\hat{\mu }}_{\alpha }^{\mathrm{sel}}=\mu_{\alpha }^{\mathrm{sel}}$ for the channel passing ions and the confined species with

(21) $$\begin{array}{cc} \mu_{\alpha }^{\mathrm{sel}}=g_{\alpha }^{\mathrm{sel}}+RT\;\ln\left(y_{\alpha }\right)-RT\;\ln\left(1-\sum_{\beta \in I^{\mathrm{pass}}\cup I^{\mathrm{conf}}}y_{\beta }\right), & \;\alpha \in I^{\mathrm{pass}}\cup I^{\mathrm{conf}}, \end{array}$$

such that the diffusional flux (Equation (8)) becomes

(22) $$\begin{array}{c} J_{\alpha }^{\mathrm{sel}}=-\sum_{\beta \in I^{j}}M_{\alpha \beta }^{j}\left(RT\;\nabla [\ln\left(y_{\beta }\right)-\ln(1-\sum_{\gamma \in I^{\mathrm{pass}}\cup I^{\mathrm{conf}}}y_{\gamma })]+Fz_{\beta }^{\mathrm{sel}}\nabla \varphi \right). \end{array}$$

We would like to point out that it is also possible that different species require a different number of lattice sites.

### 2.5. Reactions

Since ions will pass the selectivity filter in a (partially) dehydrated state, we will introduce desolvation (resolvation) reactions on the interfaces between the outer (inner) domain and the filter region. In general we will denote the reactions on an interface $S^{j,k}$ as follows

(23) $$\begin{array}{c} A_{\alpha }^{j}⇌A_{\alpha }^{k}+\left(\kappa_{\alpha }^{j}-\kappa_{\alpha }^{k}\right)A_{0}. \end{array}$$

This means that the solvated species $A_{\alpha }^{j}$ “reacts” on the interface to the desolvated species $A_{\alpha }^{k}$ and $\left(\kappa_{\alpha }^{j}-\kappa_{\alpha }^{k}\right)$ solvent molecules $A_{0}$. Thereby species $A_{\alpha }^{j}$ and the solvent molecule live in domain $\Omega^{j}$, and species $A_{\alpha }^{k}$ lives in domain $\Omega^{k}$. Note that it is possible that ions only partially dehydrate upon entering the SF, which means that, for example, $\kappa_{\alpha }^{\mathrm{out}}>\kappa_{\alpha }^{\mathrm{sel}}>0$. However, it is also possible that they dehydrate completely, so that $\kappa_{\alpha }^{\mathrm{sel}}=0$. From that reaction, we can define a surface affinity, which gives a measure of how likely the reaction will take place.

(24) $$\begin{array}{c} \;{\underset{s}{\lambda }}_{\alpha }^{j,k}:=\mu_{\alpha }^{k}{\;|}_{k}^{j,k}+\left(\kappa_{\alpha }^{j}-\kappa_{\alpha }^{k}\right)\mu_{0}\;{{|}_{j}^{j,k}-\mu_{\alpha }^{j}\;|}_{j}^{j,k}, \end{array}$$

where, for example, $\mu_{\alpha }^{k}{\;|}_{k}^{j,k}$ is the chemical potential function defined in $\Omega^{k}$ evaluated on the interface $S^{j,k}$. The reaction rates on the interfaces then depend on this surface affinity and are given as follows:

(25) $$\begin{array}{c} \;{\underset{s}{R}}_{\alpha }^{j,k}=\;{\underset{s}{L}}_{\alpha }^{j,k}\;r_{\alpha }\left(\frac{\;{\underset{s}{\lambda }}_{\alpha }^{j,k}}{RT\;}\right)\;\mathrm{with}\;r_{\alpha }\left(x\right):=e^{\beta_{\alpha }\;x}\;-e^{-(1-\beta_{\alpha })\;x}\;, \end{array}$$

where $\;{\underset{s}{L}}_{\alpha }^{j,k}$ and $\beta_{\alpha }$ are constants.

Note that the reaction constant $\;{\underset{s}{L}}_{\alpha }^{j,k}$ determines how fast the reaction will proceed. If the reaction is fast, then $\;{\underset{s}{L}}_{\alpha }^{j,k}\to \infty$, which further implies the reaction equilibrium side constraint $\;{\underset{s}{\lambda }}_{\alpha }^{j,k}=0$. In addition, we can rewrite the surface affinity given in Equation (24) such that we obtain

(26) $$\begin{array}{c} \;{\underset{s}{\lambda }}_{\alpha }^{j,k}:=\Delta g_{\alpha }^{j,k}+{\tilde{\mu }}_{\alpha }^{k}{\;|}_{k}^{j,k}+\left(\kappa_{\alpha }^{j}-\kappa_{\alpha }^{k}\right){\tilde{\mu }}_{0}\;{{|}_{j}^{j,k}-{\tilde{\mu }}_{\alpha }^{j}\;|}_{j}^{j,k}, \end{array}$$

with $\Delta g_{\alpha }^{j,k}=g_{\alpha }^{k}-g_{0}^{j}-g_{\alpha }^{j}$ and ${\tilde{\mu }}_{\alpha }^{l}=\mu_{\alpha }^{l}-g_{\alpha }^{l}$ (for l={j,k}). The quantities $g_{\alpha }^{l}$ are the reference potentials. Thus, the quantity $\Delta g_{\alpha }^{j,k}$ indicates whether an ion favors being solvated in the electrolyte or desolvated in the selectivity filter. In other words, the interface reactions become the energy barrier due to desolvation that the ions have to overcome when they enter the SF. This means that the surface affinity (Equation (24)) of this reaction thus accounts entropically as well as energetically for solvation effects. The quantities $\;{\underset{s}{L}}_{\alpha }^{j,k}$ and $\Delta g_{\alpha }^{j,k}$ need to be determined experimentally or with experimental data.

It would also be possible to include additional species that might be involved in the interface reactions, such as the free-moving but confined ions in the SF.

### 2.6. Boundary Conditions

We have to specify boundary conditions essentially at the interfaces $S^{\mathrm{out}}$, $S^{\mathrm{in}}$, $S^{\mathrm{out},\mathrm{sel}}$, $S^{\mathrm{in},\mathrm{sel}}$, and $S^{\mathrm{charge}}$. If for a boundary the conditions are not explicitly mentioned, we will assume homogeneous Neumann boundary conditions for the concentrations and the electrostatic potential and no-slip boundary conditions for the velocity.

#### 2.6.1. Exterior Boundaries

On the exterior boundaries $S^{\mathrm{out}}$ and $S^{\mathrm{in}}$, we can consider Dirichlet boundary conditions for the molar densities and the electrostatic potential.

(27) $$\begin{array}{c} n_{\alpha }^{j}{{|}^{j}={\left[n_{\alpha }\right]}^{j}=\mathrm{const}.\;\forall \alpha \in I^{j},\;\varphi |}^{j}=\varphi^{j}=\mathrm{const}., \end{array}$$

homogeneous Neumann boundary conditions

(28) $$\begin{array}{c} J_{\alpha }^{j}\;\cdot \;n{{|}^{j}=0\;\forall \alpha \in I^{j},\;\nabla \varphi \;\cdot \;n|}^{j}=0 \end{array}$$

or inhomogeneous Neumann boundary conditions, which, in the case of the concentrations, are reaction boundary conditions

(29) $$\begin{array}{c} J_{\alpha }^{j}{\;\cdot \;n|}^{j}=\;{\underset{s}{R}}_{\alpha }^{j}\left(\;{\underset{s}{\lambda }}_{\alpha }^{j}\right)\;\forall \alpha \in I^{j} \end{array}$$

and in case the electrostatic potential is a surface charge condition

(30) $$\begin{array}{c} \left[\;\left[-\varepsilon_{0}\left(1+\chi^{j}\right)\nabla \varphi \right]\;\right]{\;\cdot \;n|}^{j}=\;{\underset{s}{q}}^{j}=\mathrm{const}.. \end{array}$$

It would also be possible to apply a prescribed electrical current on one of the boundaries.

(31) $$\begin{array}{c} j_{q}^{j}{\;\cdot \;n|}^{j}=I_{q}^{j}=\mathrm{const}.. \end{array}$$

Note that “constant” refers here to constant with respect to all other state variables. However, ${\left[n_{\alpha }\right]}^{j}$, $\varphi^{j}$, or $I_{q}^{j}$ could also be time-dependent, for example. Depending on the studied experiment or problem, a mixture of different types of boundary conditions is likely. For the velocity and the pressure, we assume *do nothing* boundary conditions.

(32) $$\begin{array}{c} {\nu v\;\cdot \;n|}^{j}{-pn|}^{j}=0. \end{array}$$

Within the selectivity filter on $S^{\mathrm{charge}}$, we apply a constant surface charge via the Neumann boundary condition.

(33) $$\begin{array}{c} \left[\;\left[-\varepsilon_{0}\left(1+\chi^{\mathrm{sel}}\right)\nabla \varphi \right]\;\right]{\;\cdot \;n|}^{\mathrm{charge}}=\;{\underset{s}{q}}^{\mathrm{sel}}=\mathrm{const}., \end{array}$$

homogeneous Neumann boundary conditions for the concentrations

(34) $$\begin{array}{c} J_{\alpha }^{\mathrm{sel}}{\;\cdot \;n|}^{\mathrm{charge}}=0\;\forall \alpha \in I^{\mathrm{sel}} \end{array}$$

and no-slip for the velocity

(35) $$\begin{array}{c} {v|}^{\mathrm{charge}}=0. \end{array}$$

#### 2.6.2. Interior Boundaries

On the interior boundaries $S^{\mathrm{out},\mathrm{sel}}$ and $S^{\mathrm{in},\mathrm{sel}}$ we assume interface reactions for the species, such that

(36) $$\begin{array}{c} J_{\alpha }^{j}{\;|}_{j}^{j,\mathrm{sel}}=-J_{\alpha }^{\mathrm{sel}}\;{|}_{\mathrm{sel}}^{j,\mathrm{sel}}=\;{\underset{s}{R}}_{\alpha }^{j,\mathrm{sel}}\;\forall \alpha \in I^{j}\cup I^{\mathrm{sel}}\setminus I^{\mathrm{conf}}, \end{array}$$

with j={out,in}, $\;{\underset{s}{R}}_{\alpha }^{\mathrm{out},\mathrm{sel}}$ and $\;{\underset{s}{R}}_{\alpha }^{\mathrm{in},\mathrm{sel}}$ given by Equation (25).

For the confined species within the SF domain, we apply a no-flux boundary condition

(37) $$\begin{array}{c} J_{\alpha }^{\mathrm{sel}}{\;|}_{\mathrm{sel}}^{j,\mathrm{sel}}=0\;\forall \alpha \in I^{\mathrm{conf}}. \end{array}$$

For the electrostatic potential, the pressure, and the mass flux, we assume continuity, i.e., that they are continuous across the whole domain, which means that

(38) $$\begin{array}{c} {\varphi \;|}_{j}^{j,k}{=\varphi \;|}_{k}^{j,k}{,\;p\;|}_{j}^{j,k}{=p\;|}_{k}^{j,k},\;\rho^{j}v\;{{|}_{j}^{j,k}=\rho^{k}v\;|}_{k}^{j,k}. \end{array}$$

### 2.7. Equilibrium and General Assumptions

In the following, some assumptions are elaborated that can be applied in order to reduce the complexity of the system.

#### 2.7.1. Mechanical Equilibrium

We assume that the system is in mechanical equilibrium, such that v=0 in whole Ω. This reduces the Equations (5) and (6) to

(39) $$\begin{array}{c} \nabla p=-q^{j}\nabla \varphi . \end{array}$$

Taking the divergence of both sides yields [36]

(40) $$\begin{array}{c} \nabla \;\cdot \;\left[\nabla p+q^{j}\nabla \varphi \right]=0. \end{array}$$

#### 2.7.2. Reactions in Equilibrium

It would also be possible to assume that the interface reactions are in equilibrium. Which is, for example, the case if the reactions are fast such that $\;{\underset{s}{L}}_{\alpha }^{j,k}\to \infty$. This means we could apply the following Dirichlet boundary conditions on the interfaces $S^{\mathrm{out},\mathrm{sel}}$ and $S^{\mathrm{in},\mathrm{sel}}$ for the ion species

(41) $$\begin{array}{c} \mu_{\alpha }^{j}{|}_{j}^{j,k}-\left(\kappa_{\alpha }^{j}-\kappa_{\alpha }^{k}\right)\mu_{0}{{|}_{j}^{j,k}=\mu_{\alpha }^{k}|}_{k}^{j,k}\;\forall \alpha \in I^{j}\cup I^{k} \end{array}$$

to impose continuity in the chemical potentials. Another assumption is that the reactions are faster than diffusion and thus are negligible, which essentially means that we can impose continuity for the concentrations.

(42) $$\begin{array}{c} n_{\alpha }^{j}{{|}_{j}^{j,k}=n_{\alpha }^{k}|}_{k}^{j,k}\;\forall \alpha \in I^{j}\cup I^{k}. \end{array}$$

#### 2.7.3. Mobility Matrix

The mobility matrices $M_{\alpha \beta }$ are and can in general be functions of the thermodynamic state variables $\left(n_{0},\ldots ,n_{N}\right)$. For the sake of this work, we consider a Nernst–Einstein-type relation for the diagonal elements.

(43) $$\begin{array}{c} M_{\alpha \alpha }^{j}=\frac{D_{\alpha }^{j}}{RT\;}n_{\alpha }^{j}. \end{array}$$

The off-diagonal entries of the mobility matrix $M_{\alpha \beta }^{j}$ are chosen to be zero for this work, i.e., $M_{\alpha \beta }^{j}=0$ for α≠β.

Note that other relations, such as $M_{\alpha \alpha }^{j}=\left(D_{\alpha }^{j}/RT\;\right)n_{\alpha }^{j}\;y_{0}$ for the diagonal entries, non-zero off-diagonal entries, which lead to cross-diffusion effects, or general Maxwell-Stefan diffusion relations [37], are also imaginable, thermodynamically consistent, and straightforward with the presented model framework.

It would also be possible to consider a mobility of the form $M_{\alpha \alpha }^{\mathrm{sel}}=\left(D_{\alpha }^{\mathrm{sel}}/RT\;\right)n_{\alpha }^{\mathrm{sel}}\left(1\;-\;y_{\alpha }^{\mathrm{sel}}\right)$ within the SF to additionally account for the idea of the lattice mixing entropy.

#### 2.7.4. General Assumptions

Throughout this work we will assume that ions that enter the selectivity filter domain $\Omega^{\mathrm{sel}}$ become fully dehydrated, i.e., $\kappa_{\alpha }^{\mathrm{sel}}=0$. This essentially means that for ease of notation we can drop the index ^*j*^ for the mass, volume, number of solvent molecules, and charge numbers, such that $m_{\alpha }^{j}=m_{\alpha }$, $v_{\alpha }^{j}=v_{\alpha }$, $\kappa_{\alpha }^{j}=\kappa_{\alpha }$, and $z_{\alpha }^{j}=z_{\alpha }$.

### 2.8. Summary Equations

Applying the before-mentioned equilibrium and general assumption, we can specify the balance equations for the bulk and the selectivity filter regions. Since the quantities $\;{\underset{s}{L}}_{\alpha }^{j,k}$ and $\Delta g_{\alpha }^{j,k}$ need to be determined experimentally or with experimental data, we will consider continuous concentrations (Equation (42)) for all ions throughout this work. Hence, we will drop the superscript for this quantity due to better readability. A comprehensive study, which also includes interfacial reactions, is the subject of another planned project.

#### 2.8.1. Balance Equations

**Bulk.** In the bulk domains $\Omega^{\mathrm{out}}$ and $\Omega^{\mathrm{in}}$, we find

(44a) $$\begin{array}{c} \partial_{t}n_{\alpha }-\nabla \;\cdot \;\left[D_{\alpha }^{\mathrm{bulk}}n_{\alpha }\nabla \left(\ln\left(y_{\alpha }\right)-\frac{m_{\alpha }}{m_{0}}\ln\left(y_{0}\right)+\frac{1}{RT\;}\left(v_{\alpha }-\frac{m_{\alpha }}{m_{0}}v_{0}\right)p+\frac{F}{RT\;}z_{\alpha }\varphi \right)\right]=0, \end{array}$$

(44b) $$\begin{array}{c} -\varepsilon_{0}\left(1+\chi^{\mathrm{bulk}}\right)\Delta \varphi =q^{\mathrm{bulk}}, \end{array}$$

(44c) $$\begin{array}{c} \Delta p=-\nabla \;\cdot \;(q^{\mathrm{bulk}}\nabla \varphi ), \end{array}$$

with j=bulk={out,in}.

**Selectivity filter.** Within the selectivity filter domain $\Omega^{\mathrm{sel}}$, we only consider fully dehydrated ions. The system then becomes

(45a) $$\begin{array}{c} \partial_{t}n_{\alpha }-\nabla \;\cdot \;\left[D_{\alpha }^{\mathrm{sel}}n_{\alpha }\nabla \left(\ln\left(y_{\alpha }\right)-\ln\left(1-\sum_{\beta \in I^{\mathrm{pass}}}y_{\beta }-y_{N+1}\right)+\frac{F}{RT\;}z_{\alpha }\varphi \right)\right]=0, \end{array}$$

where $y_{N+1}$ is the mobile but confined species in the filter region,

(45b) $$\begin{array}{cc} \partial_{t}n_{N+1}-\nabla \;\cdot \; & \left[D_{N+1}^{\mathrm{sel}}n_{N+1}\nabla \left(\ln\left(y_{N+1}\right)\right.\right.\\ \; & \left.\left.\;-\ln\left(1-\sum_{\beta \in I^{\mathrm{pass}}}y_{\beta }-y_{N+1}\right)+\frac{F}{RT\;}z_{N+1}\varphi \right)\right]=0, \end{array}$$

(45c) $$\begin{array}{c} -\varepsilon_{0}\left(1+\chi^{\mathrm{sel}}\right)\Delta \varphi =q^{\mathrm{sel}}, \end{array}$$

(45d) $$\begin{array}{c} \Delta p=-\nabla \;\cdot \;(q^{\mathrm{sel}}\nabla \varphi ). \end{array}$$

#### 2.8.2. Boundary Conditions

Throughout this work, we will apply the following boundary conditions.

**Exterior boundaries.** We will consider Dirichlet boundary conditions on $S^{\mathrm{out}}$ and $S^{\mathrm{in}}$ for the molar densities, the electrostatic potential, and the pressure

(46) $$\begin{array}{cc} \; & n_{\alpha }{|}^{\mathrm{out}}={\left[n_{\alpha }\right]}^{\mathrm{out}}\;\forall \alpha \in I^{\mathrm{out}},\;\varphi {{|}^{\mathrm{out}}=\varphi^{\mathrm{out}},\;p|}^{\mathrm{out}}=p^{\mathrm{out}},\\ \; & n_{\alpha }{|}^{\mathrm{in}}={\left[n_{\alpha }\right]}^{\mathrm{in}}\;\forall \alpha \in I^{\mathrm{in}},\;\varphi {{|}^{\mathrm{in}}=\varphi^{\mathrm{in}},\;p|}^{\mathrm{in}}=p^{\mathrm{in}}. \end{array}$$

Within the selectivity filter on $S^{\mathrm{charge}}$, we apply a constant surface charge

(47) $$\begin{array}{c} \left[\;\left[-\varepsilon_{0}\left(1+\chi^{\mathrm{sel}}\right)\nabla \varphi \right]\;\right]{\;\cdot \;n|}^{\mathrm{charge}}=\;{\underset{s}{q}}^{\mathrm{sel}}=\mathrm{const}. \end{array}$$

and homogeneous boundary conditions for the concentrations and the pressure

(48) $$\begin{array}{c} J_{\alpha }^{\mathrm{sel}}\;\cdot \;n{{|}^{\mathrm{charge}}=0\;\forall \alpha \in I^{\mathrm{sel}},\;\nabla p\;\cdot \;n|}^{\mathrm{charge}}=0. \end{array}$$

**Interior boundaries.** For the electrostatic potential and the pressure, we assume continuity on the interior boundaries $S^{\mathrm{out},\mathrm{sel}}$ and $S^{\mathrm{in},\mathrm{sel}}$

(49) $$\begin{array}{c} {\varphi |}_{\mathrm{out}}^{\mathrm{out},\mathrm{sel}}{=\varphi |}_{\mathrm{sel}}^{\mathrm{out},\mathrm{sel}}{,\;p|}_{\mathrm{out}}^{\mathrm{out},\mathrm{sel}}{=p|}_{\mathrm{sel}}^{\mathrm{out},\mathrm{sel}}{,\;\varphi |}_{\mathrm{in}}^{\mathrm{in},\mathrm{sel}}{=\varphi |}_{\mathrm{sel}}^{\mathrm{in},\mathrm{sel}},\;p{{|}_{\mathrm{in}}^{\mathrm{in},\mathrm{sel}}=p|}_{\mathrm{sel}}^{\mathrm{in},\mathrm{sel}}. \end{array}$$

Throughout this work, we will also assume that the reactions are fast compared to the diffusion through the selecitivity filter domain, which means that we also consider continuity in the concentrations

(50) $$\begin{array}{c} n_{\alpha }^{\mathrm{out}}{|}_{\mathrm{out}}^{\mathrm{out},\mathrm{sel}}=n_{\alpha }^{\mathrm{sel}}{|}_{\mathrm{sel}}^{\mathrm{out},\mathrm{sel}},\;n_{\alpha }^{\mathrm{in}}{{|}_{\mathrm{in}}^{\mathrm{in},\mathrm{sel}}=n_{\alpha }^{\mathrm{sel}}|}_{\mathrm{sel}}^{\mathrm{in},\mathrm{sel}}. \end{array}$$

For the confined species within the SF domain, we apply the no-flux boundary condition

(51) $$\begin{array}{c} J_{N+1}^{\mathrm{sel}}\;{{|}_{\mathrm{sel}}^{\mathrm{out},\mathrm{sel}}=J_{N+1}^{\mathrm{sel}}\;|}_{\mathrm{sel}}^{\mathrm{in},\mathrm{sel}}=0. \end{array}$$

To incorporate the interface reactions will be the subject of future work. An illustration of the domain, including the different boundaries, is given in Figure 2.

### 2.9. Numerical Method

In order to solve the system, we use Julia and the VoronoiFVM.jl package [38], which implements the Voronoi cell-based finite volume method. The boundary conforming Delaunay triangulation of the domain Ω is generated with the help of the Triangle mesh generator [39] using the interface SimplexGridFactory.jl.

#### Discretization

The transport equations (Equations (44a) and (45a)) are discretized using a backward Euler scheme in time, which guarantees unconditional stability for the stiff problems at hand. For the flux we use a Scharfetter–Gummel-inspired discretization [40,41]. To do so, the excess chemical potential.

(52) $$\begin{array}{c} \nu_{\alpha }\left(n_{0},\ldots ,n_{N},p\right):={\hat{\mu }}_{\alpha }\left(n_{0},\ldots ,n_{N},p\right)-\ln\left(n_{\alpha }\right) \end{array}$$

is introduced to rewrite the flux term (Equation (44a)) in the bulk regions as

(53) $$\begin{array}{c} J_{\alpha }^{\mathrm{bulk}}=-D_{\alpha }^{\mathrm{bulk}}\left[\nabla n_{\alpha }+n_{\alpha }\nabla \left(\nu_{\alpha }^{\mathrm{bulk}}+\frac{F}{RT\;}z_{\alpha }\varphi \right)\right], \end{array}$$

with

(54) $$\begin{array}{c} \nu_{\alpha }^{\mathrm{bulk}}=\left(v_{\alpha }-\frac{m_{\alpha }}{m_{0}}v_{0}\right)\frac{p}{RT\;}-\frac{m_{\alpha }}{m_{0}}\ln\left(y_{0}\right)-\ln\left(n\right). \end{array}$$

In the selectivity filter region, the flux terms (Equations (45a) and (45b)) are rewritten as

(55) $$\begin{array}{c} J_{\alpha }^{\mathrm{sel}}=-D_{\alpha }^{\mathrm{sel}}\left[\nabla n_{\alpha }+n_{\alpha }\nabla \left(\nu_{\alpha }^{\mathrm{sel}}+\frac{F}{RT\;}z_{\alpha }\varphi \right)\right], \end{array}$$

with

(56) $$\begin{array}{c} \nu_{\alpha }^{\mathrm{sel}}=-\ln\left(1-\sum_{\beta \in I^{\mathrm{pass}}}y_{\beta }-y_{N+1}\right). \end{array}$$

The term $\nu_{\alpha }^{j}+\left(F/RT\;\right)z_{\alpha }\varphi$ then replaces the electrostatic potential in the classical Scharfetter-Gummel discretization ansatz.

The Poisson equations (Equations (44b) and (45c)) and the momentum balance (Equations (44c) and (45d)) are discretized using the classical two-point flux approximation.

## 3. Numerical Results for a Calcium-Selective Ion Channel

Within this work we exemplarily study a calcium-selective L-type channel. We compare the model simulations to experimental data and perform a global sensitivity analysis of the currents to identify the parameters that have the greatest influence.

In order to compare the model with experimental data, we assume that the system is in steady state, such that the partial derivatives with respect to time in Equations (44a) and (45a) vanish. In the inner and outer electrolyte $\Omega^{\mathrm{bulk}}$, we consider a mixture of sodium $A_{1}\;=\;A_{{\mathrm{Na}}^{+}}$, calcium $A_{2}=A_{{\mathrm{Ca}}^{2+}}$, and chloride $A_{3}=A_{{\mathrm{Cl}}^{-}}$ ions, as well as water molecules $A_{0}=A_{H_{2}O}$ as solvent. The dimensions of the considered domain are based on those for calcium selective L-type ${\mathrm{Ca}}_{v}$1.2 channels [34,40,42,43] and are given in Figure 2 (right). For all simulations we use a cylindrically symmetric domain with a radius of $r_{\mathrm{domain}}=25$ Å and a length of $l_{\mathrm{domain}}=40$ Å [40]. The lipid bilayer has a thickness of $d_{\mathrm{lip}}=20$ Å [40]. In the center the membrane contains the pore with a radius of r=4.5 Å at the narrowest point. The selectivity filter region within the channel has a length of l=14 Å. We do not apply the surface charge on the whole selectivity filter but rather to a smaller part since the ions mainly interact with the negatively charged EEEE (Glu-Glu-Glu-Glu) locus. The length of the applied surface charge is $l_{\mathrm{charge}}=8$ Å. Since, according to G. M. Lipkind and H. A. Fozzard [34], the side chains of the four glutamates may form a structure that can be interpreted as three binding sites, we model the selectivity filter domain $\Omega_{\mathrm{sel}}$ accordingly. In this region, we do not assume the presence of water or a mobile confined species such as oxygen. Instead, we follow the interpretation that the carboxylate groups of the EEEE locus form fixed lattice sites on which the permeating ions diffuse. Consequently, in the following studies we set $n_{N+1}=0$ and impose a fixed negative surface charge along the channel wall. A more refined treatment of this aspect is planned for future work.

**Figure 4:**
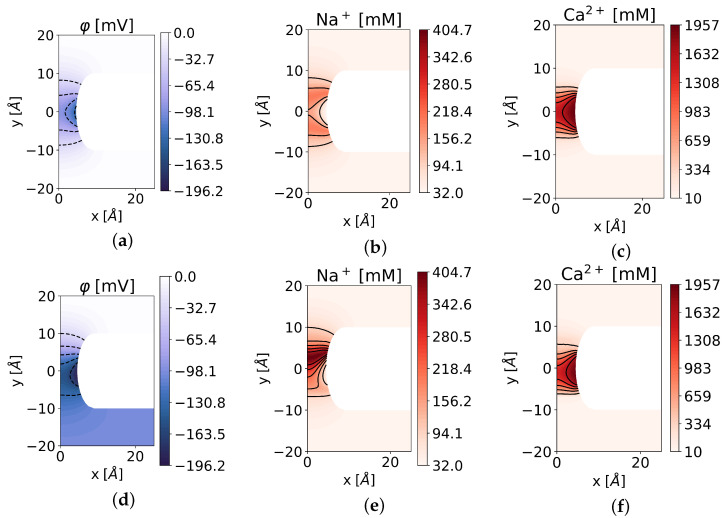
Contour plots of (**a**) the electrostatic potential, (**b**) the sodium, and (**c**) the calcium concentration for no potential difference, i.e., E=0. Contour plots of (**d**) the electrostatic potential, (**e**) the sodium, and (**f**) the calcium concentration for a potential difference of E=−20mV.

We performed a simulation for a mixture of ${\left[\mathrm{NaCl}\right]}^{\mathrm{out}}={\left[\mathrm{NaCl}\right]}^{\mathrm{in}}=32$ mM and ${\left[{\mathrm{CaCl}}_{2}\right]}^{\mathrm{out}}={\left[{\mathrm{CaCl}}_{2}\right]}^{\mathrm{in}}=10$ mM with a surface charge of $\;{\underset{s}{q}}^{\mathrm{sel}}=-1e_{0}$ $/mm^{2}$ on $S^{\mathrm{charge}}$. In a first scenario we did not apply a potential difference, i.e., $\varphi^{\mathrm{out}}=\varphi^{\mathrm{in}}=0$, such that E=0. We find that mainly calcium is present in the selectivity filter (−8≤x≤8) and close to the charged wall, even occupying almost all available lattice sites with $n_{\ell }=2$ M (Figure 4c). Closer to the symmetry axis, we find a mixture of sodium and calcium, whereby the calcium concentration is around one order of magnitude higher (Figure 4b). A look at the solution for the electrostatic potential in Figure 4a shows that the influence of the negative surface charge is not limited to the selectivity filter but also has an effect on the bulk regions.

If we now apply an additional negative potential difference with $\varphi^{\mathrm{in}}=-20$ mV, we find that the entire intracellular domain $\Omega^{\mathrm{in}}$ is negatively charged (Figure 4d). In this case, it can be observed that sodium is drawn into the channel towards the negative intracellular space, and its concentration increases in the upper part of that region (0≤x≤8) by a factor of 2 (Figure 4e). The same applies to calcium, which now mainly accumulates in the lower area of the filter (Figure 4f).

### 3.1. Comparison with Experimental Data

We compare our simulation results to an experimentally measured current by Almers et al. [14]. In this work the calcium selectivity of the pore was studied. The authors measured the total current through the pore and observed that the permeability of calcium channels depends on the ${\mathrm{Ca}}^{2+}$ concentration.

To compare the model to the data, we calculate the ionic current for a fixed applied potential difference with $\varphi^{\mathrm{out}}=0$ mV and $\varphi^{\mathrm{in}}=-20$ mV, s.t. E=−20 mV. For the mixture, we use the following concentrations: [NaCl${]}^{\mathrm{out}}=[$NaCl${]}^{\mathrm{in}}=32$ mM, [${\mathrm{CaCl}}_{2}{]}^{\mathrm{out}}=\left(5.13\;\cdot \;10^{-7}-13.18\right)$ mM and [${\mathrm{CaCl}}^{2}{]}^{\mathrm{in}}=0$ M. Within the selectivity filter a permanent surface charge is applied with $\;{\underset{s}{q}}^{\mathrm{sel}}=-2\;e_{0}$ $/nm^{2}$ and a number of lattice sites of $n_{\ell }=2$ M is considered. For the cations we derive different diffusion coefficients within the selectivity filter, with $D_{{\mathrm{Ca}}^{2+}}^{\mathrm{sel}}=2.5\times 10^{-4}\times D_{{\mathrm{Ca}}^{2+}}^{\mathrm{bulk}}$ and $D_{{\mathrm{Na}}^{+}}^{\mathrm{sel}}=1.5\times 10^{-3}\times D_{{\mathrm{Na}}^{+}}^{\mathrm{bulk}}$. All other relevant parameter values are given in Table A1. The diffusion coefficients, the surface charge, and the number of lattice sites within the selectivity filter domain were determined by fitting the model to the experimental data from Almers et al. [14]. We would like to point out that the number of lattice sites is actually determined by the structure of the SF. However, it is unclear how exactly this can be translated into the continuum model, which is why we treat the parameter $n_{\ell }$ as a fitting parameter. The determined diffusion coefficients within the selectivity filter are some orders of magnitude smaller than those proposed by MD simulations and other continuum models. Here, diffusion coefficients were determined that are 5–10 times smaller than in the bulk [40,44,45,46,47]. Nevertheless, one has to be careful when comparing these results; for example, Allen et al. [44] study KcsA potassium channels with a length of 40 Å, while our channel has a length of 20 Å. Mamonov et al. study the diffusion of $K^{+}$ in the Gramacidin A channel and vary the dielectric constant within the selectivity filter, while we keep the dielectric properties constant throughout the whole domain, i.e., we choose $\varepsilon_{r}^{\mathrm{sel}}=\varepsilon_{r}^{\mathrm{bulk}}$. It is important to mention that the relative permittivity within the SF range is most likely smaller than in the bulk, and that this aspect should be taken into account, especially if one is interested in determining more realistic values for unknown parameters. In this case, however, we recommend considering a suitable parameter estimation method to prevent over- or underfitting of the model. The primary goal of this work was to develop a model framework that can be applied to a variety of channels and problems, rather than to derive specific parameter values. This particular example was chosen to show that the model is capable of representing complex phenomena such as the anomalous molar fraction effect. However, we elaborate the influence of $\varepsilon_{r}^{\mathrm{sel}}\neq \varepsilon_{r}^{\mathrm{bulk}}$ within the following parameter study.

**Figure 5:**
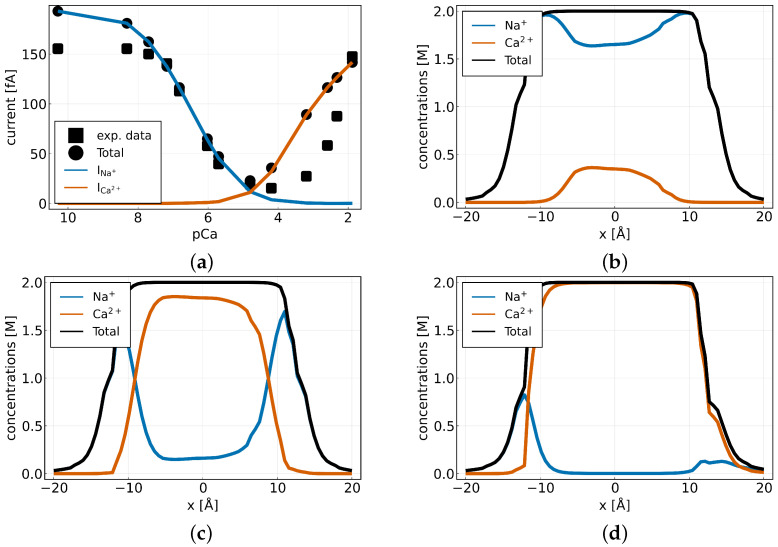
(**a**) Total current for different calcium concentrations in the outer bath. Model simulation (circles) compared to experimental data (squares) from Almers et al. Figure 11A [14]. Sodium current (blue) and calcium current (red-orange) for different calcium concentrations pCa = $-\log_{10}\left({\left[{\mathrm{CaCl}}_{2}\right]}^{\mathrm{out}}\right)$ in the outer bath. (**b**–**d**) Sodium (blue), calcium (red-orange) and total (black) concentrations along the *y*-axis at x=0. For an extracellular calcium concentration of (**a**) pCa = 7.18, i.e., ${\left[{\mathrm{CaCl}}_{2}\right]}^{\mathrm{out}}=6.6\;\cdot \;10^{-2}$ mM, (**b**) pCa = 4.8, i.e., ${\left[{\mathrm{CaCl}}_{2}\right]}^{\mathrm{out}}=0.016$ mM and (**c**) pCa = 2.3, i.e., ${\left[{\mathrm{CaCl}}_{2}\right]}^{\mathrm{out}}=4.79$ mM.

Figure 5a shows the comparison of the total ionic current (circles) calculated from the simulation compared to the experimentally measured total current (squares). We find that the model is in agreement with the data. The external calcium concentration is given in pCa = $-\log_{10}\left({\left[{\mathrm{CaCl}}_{2}\right]}^{\mathrm{out}}\right)$. The partial sodium ($I_{{\mathrm{Na}}^{+}}$) and calcium ($I_{{\mathrm{Ca}}^{2+}}$) currents are denoted by the blue and the red-orange lines, respectively. It illustrates that the channel is blocked to monovalent cations at a certain extracellular ${\mathrm{Ca}}^{2+}$ concentration and that the current is then dominated by the ${\mathrm{Ca}}^{2+}$ current. This behavior is also known as the anomalous mole fraction effect (AMFE). The AMFE refers to the unexpected decrease in ionic conductivity that occurs when a small quantity of a divalent ion (such as ${\mathrm{Ca}}^{2+}$) is mixed with a monovalent ion (such as ${\mathrm{Na}}^{+}$), despite both ions conducting well individually. This non-linear behavior is due to space-charge competition and ion–ion interactions within the narrow pore of the channel, which results in reduced ion flux at intermediate concentrations [33,48].

Taking a look at the sodium (blue), calcium (red-orange), and total (black) concentrations along the *y*-axis at x=0 for an extracellular calcium concentration of pCa = 7.18, i.e., ${\left[{\mathrm{CaCl}}_{2}\right]}^{\mathrm{out}}=6.6\;\cdot \;10^{-2}$ mM (Figure 5b), we find that mainly sodium is present in the channel region (−10≤x≤10). This is expected, as we also observe a high sodium current in this particular case. Particularly noteworthy is the scenario in which the two current curves intersect, indicating approximately equal values. Plotting the concentrations for pCa = 4.8, i.e., ${\left[{\mathrm{CaCl}}_{2}\right]}^{\mathrm{out}}=0.016$ mM (Figure 5c) shows that mainly calcium is present in the selectivity filter region (−8≤x≤8). However, the sodium concentration is higher in the bulk, and at the entry of the channel, both concentrations are equal. Picking a calcium concentration of pCa = 2.3, i.e., ${\left[{\mathrm{CaCl}}_{2}\right]}^{\mathrm{out}}=4.77$ mM (Figure 5d), where a high calcium current is observed, indicates that calcium exclusively occupies the filter region and fully saturates the available $n_{\ell }=2$ M space. In all three scenarios the total concentration inside the selectivity filter remains constant at 2 M, which is exactly the total number of lattice sites available.

### 3.2. Sensitivity Analysis

In a next step, we performed a global sensitivity analysis using the Sobol method [49,50,51]. To perform the analysis numerically, we used the Julia package GlobalSensitivity.jl [52] and calculated the first order and total indices for the sodium and the calcium currents. We varied the parameters $\;{\underset{s}{q}}^{\mathrm{sel}},n_{\ell },D_{{\mathrm{Ca}}^{2+}}^{\mathrm{sel}},D_{{\mathrm{Na}}^{+}}^{\mathrm{sel}},{\tilde{m}}_{{\mathrm{Ca}}^{2+}},{\tilde{m}}_{{\mathrm{Na}}^{+}}$, $\varepsilon_{r}^{\mathrm{sel}}$, which are the surface charge, the number of lattice sites, the diffusion coefficients of calcium and sodium within the selectivity filter, and the mass of calcium and sodium, respectively. We acknowledge that changing ion masses is not meaningful from an experimental perspective. However, sensitivity analysis is a valuable tool for assessing whether a parameter affects the solution and whether it is worth exploring further. For the analysis, we used the following bounds

$$\begin{array}{cc} -1.9e_{0} & \leq \;{\underset{s}{q}}^{\mathrm{sel}}\leq 0\;\left[nm^{-2}\right]\\ 1.5 & \leq n_{\ell }\leq 4\;\left[M\right]\\ 2\;\cdot \;10^{-16} & \leq D_{{\mathrm{Ca}}^{2+}}^{\mathrm{sel}}\leq 2\;\cdot \;10^{-12}\;\left[m^{2}s^{-1}\right]\\ 2\;\cdot \;10^{-15} & \leq D_{{\mathrm{Ca}}^{2+}}^{\mathrm{sel}}\leq 2\;\cdot \;10^{-11}\;\left[m^{2}s^{-1}\right]\\ 40.078\;\cdot \;10^{-2} & \leq {\tilde{m}}_{\mathrm{Ca}}^{2+}\leq 40.078\;\cdot \;10^{2}\;\left[g{\mathrm{mol}}^{-1}\right]\\ 22.989\;\cdot \;10^{-2} & \leq {\tilde{m}}_{\mathrm{Na}}^{+}\leq 22.989\;\cdot \;10^{2}\;\left[g{\mathrm{mol}}^{-1}\right]\\ 60 & \leq \varepsilon_{r}^{\mathrm{sel}}\leq \end{array}$$

and a sample size of 500 to generate the design matrices. We also conducted the analysis with a sample size of 100, but since nothing changed compared to the 500, we decided not to increase the sample size any further. We calculated the Sobol indices for the sodium and calcium current for a potential of E=−20 mV and a mixture with pCa = 4.8.

**Figure 6:**
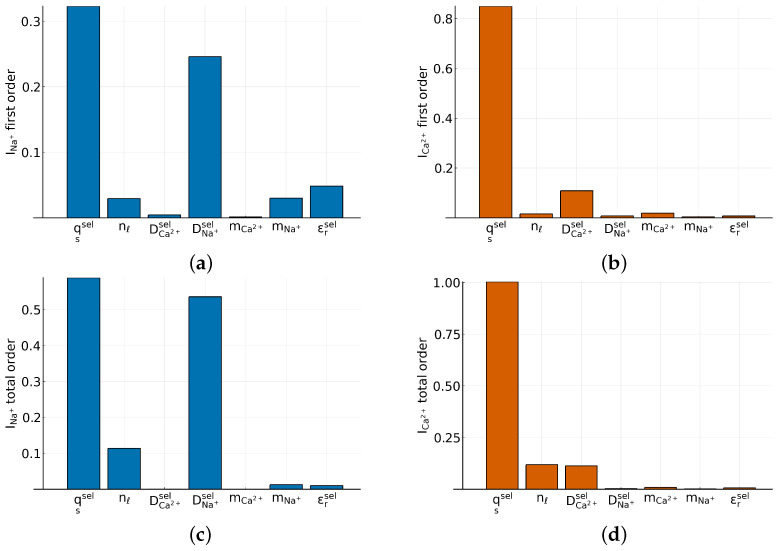
(**a**) First order Sobol indices of the sodium current, (**b**) total order Sobol indices of the sodium current, (**c**) first order Sobol indices of the calcium current and (**d**) total order Sobol indices of the calcium current.

The variance of the sodium current is dominated by the sodium diffusion coefficient and the surface charge. Both parameters exhibit substantial first-order effects (Figure 6a) and also contribute through interactions with other parameters (total order) (Figure 6d). In contrast, variations in calcium mass and in calcium diffusion coefficient do not appreciably increase either the first-order or total-order sensitivity indices. The sodium mass and the relative permittivity show small increases in first-order indices, but their total-order indices remain minor, indicating negligible interaction effects. The number of lattice sites influences output variance primarily through interactions with other parameters.

For the calcium current, surface charge is the single most influential parameter, both alone (first-order indice; Figure 6b) and in combination with others (total-order indices; Figure 6d). The calcium diffusion coefficient and the number of lattice sites also contribute to variance, both individually and via interactions. Calcium and sodium mass has no measurable effect. Changing the relative permittivity does not noticeably affect the calcium current.

### 3.3. Parameter Study

**Figure 7:**
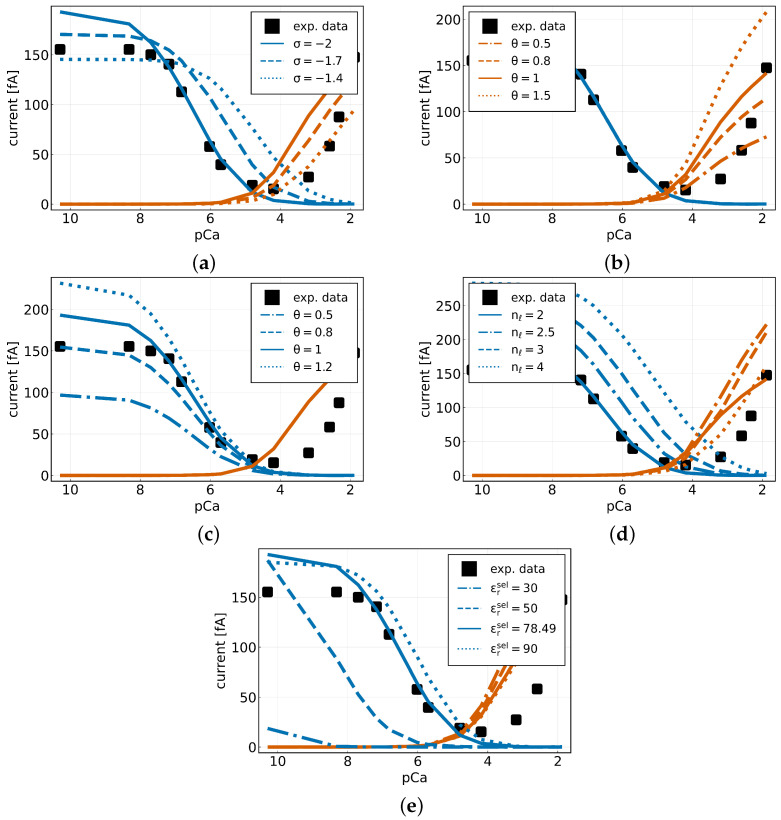
Sodium currents (blue) and calcium currents (red-orange) (**a**) for different channel surface charges with $\;{\underset{s}{q}}^{\mathrm{sel}}=\sigma e_{0}$, (**b**) for different diffusion coefficients of ${\mathrm{Ca}}^{2+}$ in the selectivity filter with $D_{{\mathrm{Ca}}^{2+}}^{\mathrm{sel},\mathrm{new}}\;=\;\theta D_{{\mathrm{Ca}}^{2+}}^{\mathrm{sel},\mathrm{old}}$, (**c**) for different diffusion coefficients of ${\mathrm{Na}}^{+}$ in the selectivity filter with $D_{{\mathrm{Na}}^{+}}^{\mathrm{sel},\mathrm{new}}=\theta D_{{\mathrm{Na}}^{+}}^{\mathrm{sel},\mathrm{old}}$, (**d**) for different lattice sites $n_{\ell }$ and (**e**) for a varying relative permittivity $\varepsilon_{r}^{\mathrm{sel}}$ in the selectivity filter.

We now repeated the experiment from before where we calculated the current for varying calcium concentrations in the extracellular domain, but this time for different parameter values for $\;{\underset{s}{q}}^{\mathrm{sel}},n_{\ell },D_{{\mathrm{Ca}}^{2+}}^{\mathrm{sel}},D_{{\mathrm{Na}}^{+}}^{\mathrm{sel}},\varepsilon_{r}^{\mathrm{sel}}$. We will not consider the mass, as it does not seem to have any significant impact on the currents. Figure 7a shows the sodium and calcium currents for different surface charges. The data indicates a decline in both the peak $I_{{\mathrm{Na}}^{+}}$ current and the peak $I_{{\mathrm{Ca}}^{2+}}$ current at specific points within the pCa range. Furthermore, a reduction in the surface charge by a factor of 0.8 (0.9) results in a decrease in both currents by a factor of 0.8 (0.9). Furthermore, a rightward shift towards higher ${\mathrm{Ca}}^{2+}$ concentrations can be observed in both curves. This finding indicates that, in the case of lower surface charge, a higher calcium concentration is necessary to achieve the blocking of sodium.

Enhancing the calcium diffusion within the selectivity filter domain by a factor of 1.5 leads to an increase of the peak $I_{{\mathrm{Ca}}^{2+}}$ by a factor of 1.5 (Figure 7b). A decrease in the mobility by 0.5 leads to a decrease in the peak calcium current by a factor of 0.5. However, as already observed in the sensitivity analysis, varying the calcium mobility has no effect on the sodium current.

As shown in Figure 7c, the sodium current is proportional to the diffusion coefficient of sodium. An increase (decrease) in the sodium mobility within the selectivity filter leads to an increase (decrease) in the peak $I_{{\mathrm{Na}}^{+}}$ and does not influence the permeation of calcium.

Furthermore, we also varied the number of available lattice sites $n_{\ell }$ (Figure 7d). As shown, when increasing the number of lattice sites the peak sodium and calcium current also increase. Thus, doubling the number of lattice sites leads to an increase in the maximum sodium current by a factor of 1.5, while it increases the maximum calcium current by a factor of 1.3. Again, we see a right shift in both current curves, indicating that as soon as more lattice sites are available, a higher calcium concentration is needed to block sodium.

We find that varying the relative permittivity within the selectivity filter mainly influences the sodium current but not significantly the calcium current (Figure 7e). For a decreasing value, the sodium current decreases. For example, considering a value of $\varepsilon_{r}^{\mathrm{sel}}=1+\chi^{\mathrm{sel}}=30$ leads to a reduction in the maximum sodium current by one order of magnitude. While the maximum calcium current only slightly increases for decreasing $\varepsilon_{r}^{\mathrm{sel}}$.

As shown in the parameter study and sensitivity analysis, the diffusion coefficients do not impact both currents simultaneously. However, the surface charge and the number of available lattice sites do have an effect. Therefore, the influence of these two parameters was examined in more detail.

**Figure 8:**
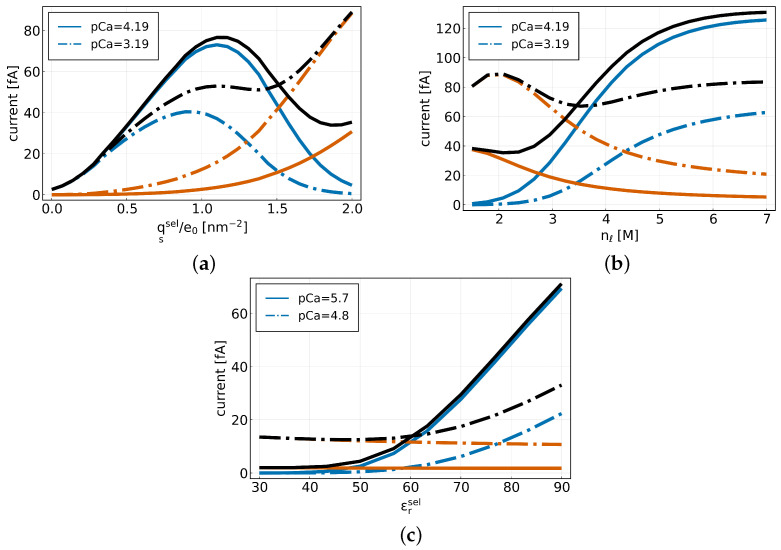
(**a**) Sodium (blue), calcium (red-orange), and total (black) currents plotted as a function of the surface charge for different calcium concentrations pCa = 4.19 (solid) and pCa = 3.19 (dash). (**b**) Sodium (blue), calcium (red-orange) and total (black) currents plotted as a function of the number of lattice sites for different calcium concentrations. (**c**) Sodium (blue), calcium (red-orange) and total (black) currents plotted as a function of the relative permittivity in the SF domain for different calcium concentrations pCa = 5.7 (solid) and pCa = 4.8 (dash).

Figure 8a shows the sodium (blue), calcium (red-orange), and total currents (black) as a function of the surface charge $\;{\underset{s}{q}}^{\mathrm{sel}}$ at a constant number of lattice sites of $n_{\ell }=2$ M and for $\varepsilon_{r}^{\mathrm{sel}}=\varepsilon_{r}^{\mathrm{bulk}}$. We performed this simulation for two different calcium concentrations, pCa = 4.19 and pCa = 3.19, at which sodium was already blocked. In both cases, we observe that a decrease in surface charge leads to a reduction in calcium current and an increase in sodium current. As the surface charge increases, the calcium current rises steadily, whereas the sodium current initially increases, reaches a maximum, and then begins to decline.

Taking a closer look exemplarily at the currents for a calcium concentration of pCa = 3.19, we find that the sodium current reaches a maximum at $\;{\underset{s}{q}}^{\mathrm{sel}}=-0.89e_{0}$ $nm^{-2}$ and the total current has a minimum at $\;{\underset{s}{q}}^{\mathrm{sel}}=-1.37e_{0}$ $nm^{-2}$.

In Figure 8b the current is plotted as a function of the lattice sites $n_{\ell }$ with a constant surface charge of $\;{\underset{s}{q}}^{\mathrm{sel}}=-2e_{0}$ $nm^{-2}$, for $\varepsilon_{r}^{\mathrm{sel}}=\varepsilon_{r}^{\mathrm{bulk}}$ for different calcium concentrations pCa = 4.19 and pCa = 3.19. We find that for an increasing number of lattice sites, the calcium current decreases while the sodium current increases.

Looking exemplarily at the curves for pCa = 3.19, it can be observed that the total current has a minimum for $n_{\ell }=3.5$ M but only slightly increases as the number of lattice sites increases. Furthermore, we find that the sodium current reaches a saturation for large $n_{\ell }$.

Plotting the current as a function of the relative permittivity in the SF for pCa = 5.7 and pCa = 4.8 illustrates that also $\varepsilon_{r}^{\mathrm{sel}}$ does not impact both currents simultaneously. The simulations were performed for constant $n_{\ell }=2$ M and $\;{\underset{s}{q}}^{\mathrm{sel}}=-2e_{0}$ $nm^{-2}$. While the sodium current increases for an increasing permittivity, the calcium current stays almost constant (Figure 8c).

This parameter study demonstrated that the process of switching between measured sodium and calcium currents depends not only on the calcium concentration but also on the surface charge and the available space within the selectivity filter, while the relative permittivity mainly influences the sodium current. For example, the increase in surface charge leads to an increase in sodium current and also in calcium current. However, the higher calcium permeability then leads to a reduction of sodium in the SF.

**Figure 9:**
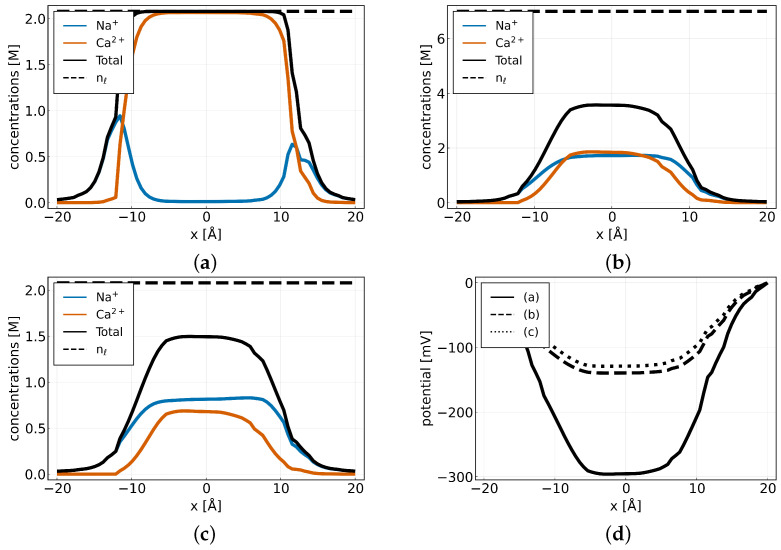
Sodium (blue), calcium (red) and total (black) cocnentrations along the *y*-axis at x=0 for (**a**) $n_{\ell }=2$ M and $\;{\underset{s}{q}}^{\mathrm{sel}}=-2e_{0}$$nm^{-2}$, (**b**) and $\;{\underset{s}{q}}^{\mathrm{sel}}=-2e_{0}$$nm^{-2}$,$n_{\ell }=7$ M (**c**) $\;{\underset{s}{q}}^{\mathrm{sel}}=-0.89e_{0}$$nm^{-2}$, $n_{\ell }=2$ M. (**d**) The elctrostatic potential plottet along the *y*-axis at x=0 for the three cases (a) (solid line), (b) (dashed line) and (c) (dotted line).

In addition to the currents, we plotted the sodium (blue) and calcium (red-orange) concentrations along the *y*-axis at x=0 in Figure 9 for different parameter combinations, $\varepsilon_{r}^{\mathrm{sel}}=\varepsilon_{r}^{\mathrm{bulk}}$ and pCa = 3.19. As in the previous experiment, we find that for the same values, i.e., $\;{\underset{s}{q}}^{\mathrm{sel}}=-2e_{0}$ $nm^{-2}$, and $n_{\ell }=2$ M, the selectivity filter (−8≤x≤8) is mainly filled with calcium. However, examining a number of lattice sites at which the sodium current is at its maximum ($\;{\underset{s}{q}}^{\mathrm{sel}}=-2e_{0}$ $nm^{-2}$, $n_{\ell }=7$ M), we observe that the sodium and calcium concentrations are almost equal within the selectivity filter region. Furthermore, we find that total concentration in this region is less than the maximum number of available lattice sites. Nevertheless, the sodium bulk concentration close to the channel remains higher than the calcium concentration which might explain the higher sodium current we measure. A similar pattern emerges with a surface charge of $\;{\underset{s}{q}}^{\mathrm{sel}}=-0.89e_{0}$ $nm^{-2}$, $n_{\ell }=2$ M, where the sodium current exceeds the calcium current and the total concentration is lower than $n_{\ell }$. In this case, the sodium concentration is slightly higher than the calcium concentration in the selectivity filter region but significantly higher in the bulk regions close to the channel. In addition, we also plotted the electrostatic potential for the three different cases (a) $n_{\ell }=2$ M and $\;{\underset{s}{q}}^{\mathrm{sel}}=-2e_{0}$ $nm^{-2}$, (b) and $\;{\underset{s}{q}}^{\mathrm{sel}}=-2e_{0}$ $nm^{-2}$, $n_{\ell }=7$ M (c) $\;{\underset{s}{q}}^{\mathrm{sel}}=-0.89e_{0}$ $nm^{-2}$, $n_{\ell }=2$ M (Figure 9d). In the event of the highest surface charge being combined with the lowest number of lattice sites (a), it is established that the potential increases to a maximum of −300 mV within the selectivity filter. This finding indicates that not all of the charge is screened by the ions. However, an increase in the number of lattice sites (b) or a decrease in the charge (c) results in a reduction of the electrostatic potential, thereby suggesting that a greater proportion of the surface charge is screened by the ions.

In addition to the currents, we plotted the sodium (blue) and calcium (red-orange) concentrations along the *y*-axis at x=0 in Figure 9 for different parameter combinations, $\varepsilon_{r}^{\mathrm{sel}}=\varepsilon_{r}^{\mathrm{bulk}}$ and pCa = 3.19. As in the previous experiment, we find that for the same values, i.e., $\;{\underset{s}{q}}^{\mathrm{sel}}=-2e_{0}$ $nm^{-2}$, and $n_{\ell }=2$ M, the selectivity filter (−8≤x≤8) is mainly filled with calcium. However, examining a number of lattice sites at which the sodium current is at its maximum ($\;{\underset{s}{q}}^{\mathrm{sel}}=-2e_{0}$ $nm^{-2}$, $n_{\ell }=7$ M), we observe that the sodium and calcium concentrations are almost equal within the selectivity filter region. Furthermore, we find that total concentration in this region is less than the maximum number of available lattice sites. Nevertheless, the sodium bulk concentration close to the channel remains higher than the calcium concentration, which might explain the higher sodium current we measure.

A similar pattern emerges with a surface charge of $\;{\underset{s}{q}}^{\mathrm{sel}}=-0.89e_{0}$ $nm^{-2}$, $n_{\ell }=2$ M, where the sodium current exceeds the calcium current and the total concentration is lower than $n_{\ell }$. In this case, the sodium concentration is slightly higher than the calcium concentration in the selectivity filter region but significantly higher in the bulk regions close to the channel.

In addition, we also plotted the electrostatic potential for the three different cases: (a) $n_{\ell }=2$ M and $\;{\underset{s}{q}}^{\mathrm{sel}}=-2e_{0}$ $nm^{-2}$, (b) $\;{\underset{s}{q}}^{\mathrm{sel}}=-2e_{0}$ $nm^{-2}$, $n_{\ell }=7$ M, and (c) $\;{\underset{s}{q}}^{\mathrm{sel}}=-0.89e_{0}$ $nm^{-2}$, $n_{\ell }=2$ M (Figure 9d). In the event of the highest surface charge being combined with the lowest number of lattice sites (a), it is established that the potential increases to a maximum of −300 mV within the selectivity filter. This finding indicates that not all of the charge is screened by the ions. However, an increase in the number of lattice sites (b) or a decrease in the charge (c) results in a reduction of the electrostatic potential, thereby suggesting that a greater proportion of the surface charge is screened by the ions.

Sodium (blue), calcium (red-orange), and total (black) concentrations along the *y*-axis at x=0 for (a) $n_{\ell }=2$ M and $\;{\underset{s}{q}}^{\mathrm{sel}}=-2e_{0}$ $nm^{-2}$, (b) and $\;{\underset{s}{q}}^{\mathrm{sel}}=-2e_{0}$ $nm^{-2}$, $n_{\ell }=7$ M, and (c) $\;{\underset{s}{q}}^{\mathrm{sel}}=-0.89e_{0}$ $nm^{-2}$, $n_{\ell }=2$ M. (d) The electrostatic potential plotted along the *y*-axis at x=0 for the three cases (a) (solid line), (b) (dashed line), and (c) (dotted line).

The parameter study showed that the blocking of sodium in calcium-selective channels does not only depend on the calcium concentration but also on the electrostatic forces and the available space inside the channel. The representation of the current as a function of these two parameters also shows a dip in the conductance, although not as strong as for the mole fractions.

## 4. Discussion and Conclusions

In this work, we have developed a thermodynamically consistent continuum model to study ion transport through ion channels under different conditions. By incorporating size-exclusion effects, desolvation, and electrostatic interactions within a modified Poisson-Nernst-Planck-Stokes framework, the model captures important physical mechanisms relevant to ion selectivity. Our simulations reproduce the characteristic current–voltage behavior observed in experiments and shed light on how channel properties and extracellular calcium concentrations influence conductivity.

Treating the selectivity filter as an embedded domain enables a physically consistent derivation of different chemical potential functions, dielectric properties, and mobilities that are region-specific. To incorporate the binding sites within the filter, we assume that they behave like a polymeric electrolyte in which immobile species, i.e., amino acid residues, form a scaffold on which the mobile channel passing ions (or within the SF confined ions) can diffuse.

Within this work we did not consider confined ions, but this aspect will be taken into account in future studies.

As it was done in previous modeling approaches, such as by Horng et al. [35], where the structural charges of the selectivity filter ${\mathrm{Ca}}_{v}$ were included by introducing confined oxygen ions.

These ions were assumed to move freely within the selectivity filter but not to enter the two baths. The confinement was modeled by homogeneous Neumann boundary conditions or a hard-wall potential [20,43,53]. Liu and Eisenberg [19,53] developed a model where they treated the free-moving oxygen ions as multiple additional binding domains. Algebraic equations must therefore be solved to calculate the electrostatic potential and the steric potential within these domains. The resulting electrostatic potential is then coupled with the PNPB system through a Dirichlet boundary condition.

Different approaches, such as DFT [20,21] or MSA [54], were used to derive the chemical potential for the species within the selectivity filter and the binding selectivity.

The presented model enables the study of the influence of different chemical potentials on the ion channel dynamics. For example, it would also be possible to consider moving lattice sites within the SF or a pressure dependency in the mixing entropy.

Solvation effects are often described implicitly by different dielectric properties within the different phases [33,43]. Therefore, the dielectric constant is often treated as a function of space [25]. Our model framework allows us to treat the relative permittivity as a piecewise constant that can be varied within the different phases. Within our framework solvation is incorporated by taking into account the solvation shell and within the interface reactions. The barrier that ions must overcome due to desolvation reactions as they enter the SF is determined by the reference chemical potentials and the reaction constants.

A first comparison with experimental data shows good agreement.

Our model reproduces the measured total current in a calcium channel for varying ${\mathrm{Ca}}^{2+}$ concentrations in the extracellular bath. The simulations show that for low calcium concentrations the channel is conductive for sodium. Moreover, our model is able to capture the AMFE that occurs due to a competition between sodium and calcium.

The parameter study depicts that mobility and electrostatic forces do influence the ion current. Furthermore, we find that the current is proportional to the diffusion coefficients and the surface charge within the channel. The global sensitivity analysis shows that the diffusion coefficients do only influence the current of the respective species and have no impact on the other species in the mixture.

Changing the relative permittivity $\varepsilon_{r}^{\mathrm{sel}}=1+\chi^{\mathrm{sel}}$ within the SF mainly impacts the sodium current. While the sodium current decreases for a decreasing permittivity, the calcium current stays almost constant.

However, we find that in addition to the calcium concentration, the surface charge in the selectivity filter region and the number of available lattice sites also impact the behavior of two cations in the mixture.

The investigation of the currents as a function of the surface charge shows that the selectivity between divalent calcium and monovalent sodium ions depends on the electric field within the selectivity filter. While the calcium current increases with increasing charge, the sodium current first increases but then drops again as the ${\mathrm{Ca}}^{2+}$ current becomes larger.

Furthermore, plotting the current as a function of the lattice sites also shows that the selectivity depends on this parameter. While the sodium current increases for an increasing number of lattice sites, the calcium current decreases simultaneously.

This is consistent with the statement that the selectivity depends on both size-exclusion effects and on the electrostatic forces [43,55].

Another example of calcium-selective ion channels are ryanodine receptors (RyRs). Wei et al. [56] and Gillespie et al. [45] found that the selectivity in RyR1, for example, also depends mainly on the electric field formed by the negatively charged residues rather than on the desolvation of the ions or other physical phenomena.

Other proposed models provide similar qualitative results as our model. Nevertheless, our approach provides a consistent description of the selectivity filter within the continuum formulation, which allows for a straightforward numerical implementation of the system. Furthermore, many models do not explicitly take dehydration of ions into account when they enter the selectivity filter.

Throughout the simulations we assumed that interface reactions are fast compared to the diffusion such that the ion concentration is continuous. In a future study we want to include interface reactions and investigate their influence on ion flux and selectivity of the channel. The aim is to find a suitable experiment that makes it possible to distinguish between diffusion and reaction-limited transport through ion channels.

The selectivity of nanopores also plays an important role in technological applications such as water treatment and desalination [57]. Here, the knowledge of biological ion channels is used to design artificial pores. It has been shown that the following characteristics have an influence on selectivity: dielectric exclusion, pore length, pore size, and the binding sites [57]. These are further aspects that could be taken into account in a parameter study by changing the length of the selectivity filter, varying the length and position of the surface charge, or changing the diameter of the pore.

In addition, our model framework allows a systematic extension to include viscoelastic properties of the ion channel protein and its coupling to the lipid bilayer, cytoplasm, and the extracellular matrix (ECM) to capture the deformation, in particular of the SF domain, as ions pass through the channel. While this has been investigated for mechanosensitive channels such as MscL- and MscS-channels (see e.g., Zhu et al. [58]), or the TRP family of channels, where mechanosensitivity and ion selectivity combine. The impact of elastic stretch of the ion channel on the ${\mathrm{Ca}}^{2+}$ flux through the protein of the type considered here is much more complex. (see e.g., Izu et al. [59]) and is the topic of our future studies.

We would like to point out that this study assumed that the channel is in an open state. However, depending on the problem being investigated, the gating mechanism should be taken into account.

The presented model framework in its current form allows the study of ion movement through nanopores under different conditions, such as different channel properties and bulk concentrations. It enables easily changing constituents within the electrolyte and their chemical properties within different regions by including different chemical potentials. Through a consistent coupling of diffusion and the incompressibility of the electrolyte, it is possible to include ions of different sizes in the model.

Our model provides a tool for the analytical and numerical investigation of parameter dependencies. By applying different types of boundary conditions, a variety of different numerical experiments can be performed, such as cyclic voltammetry or impedance measurements.

## Data Availability

The data for Figure 4, Figure 5, Figure 6, Figure 7, Figure 8 and Figure 9 are available in the Supplementary Information of this article. The code will be made available on request.

## Supplementary Materials

The following supporting information can be downloaded at: https://www.mdpi.com/article/10.3390/e27090981/s1,

## Abbreviations

The following abbreviations are used in this manuscript:

PNP	Poisson–Nernst–Planck
PNPB	Poisson–Nernst-Planck–Bikerman
IV	current–voltage
AMFE	anomalous mole fraction effect
PDE	partial differential equation

## Appendix A. Derivation of Chemical Potential Functions

### Appendix A.1. Liquid Electrolyte

The free energy density $\rho \psi^{j},j=\left\{\mathrm{in},\mathrm{out}\right\}$ for the liquid electrolyte can then be written as

(A1) $$\begin{array}{c} \rho \psi^{j}\left(T,n_{0},\ldots ,n_{N},E\right)=\rho \psi^{j,\mathrm{pol}}+\rho \psi^{j,\mathrm{mech}}+\rho \psi^{j,\mathrm{mix}}+\rho \psi^{j,\mathrm{ref}}, \end{array}$$

where

(A2) $$\begin{array}{c} \rho \psi^{j,\mathrm{pol}}=-\frac{1}{2}\varepsilon_{0}\chi^{j}{\left|E\right|}^{2} \end{array}$$

is the contribution due to polarization of the mixture,

(A3) $$\begin{array}{c} \rho \psi^{j,\mathrm{mech}}=\left(K^{j}-p^{R}\right)\left(1-H^{j}\right)+K^{j}H^{j}\ln\left(H^{j}\right), \end{array}$$

with $H^{j}=\sum_{\alpha \in I^{j}}v_{\alpha }^{j}n_{\alpha }$ and the bulk modulus $K^{j}$, is the mechanical contribution,

(A4) $$\begin{array}{c} \rho \psi^{j,\mathrm{mix}}=RT\;\sum_{\alpha \in I^{j}}n_{\alpha }\ln\left(y_{\alpha }\right), \end{array}$$

with $y_{\alpha }:=n_{\alpha }/n$ is the free energy contribution due to the entropy of mixing, and

(A5) $$\begin{array}{c} \rho \psi^{j,\mathrm{ref}}=\sum_{\alpha \in I^{j}}g_{\alpha }^{j}\;n_{\alpha }, \end{array}$$

with $g_{\alpha }^{j}=\mathrm{const}.$ is the reference state contribution. In order to calculate the chemical potential function we have to calculate

(A6) $$\begin{array}{c} \mu_{\alpha }^{j}:=\frac{\partial (\rho \psi^{j})}{\partial n_{\alpha }},\;\forall \alpha \in I^{j}. \end{array}$$

Which gives in the in-compressible limit $K^{j}\to \infty$ the chemical potential function given by Equation (11). A detailed derivation of the chemical potential is given in [28,32].

### Appendix A.2. Polymeric Electrolyte

In case of a polymeric electrolyte such as in the selectivity filter we focus on a rather simplistic material function for the free energy density $\rho \psi^{\mathrm{sel}}$, since the scope of this work is to show its general impact. In subsequent studies we plan to discuss various material models for $\rho \psi^{\mathrm{sel}}$ and show its impact on the overall behavior of the ion channel.

We consider a free energy density $\rho \psi^{\mathrm{sel}}=\rho \psi^{\mathrm{sel},\mathrm{ref}}+\rho \psi^{\mathrm{sel},\mathrm{mix}}+\rho \psi^{\mathrm{sel},\mathrm{mech}}$ with reference contribution of the pure substances $\rho \psi^{\mathrm{sel},\mathrm{ref}}$, mixing entropy contribution $\rho \psi^{\mathrm{sel},\mathrm{mix}}$ and mechanical contribution $\rho \psi^{\mathrm{sel},\mathrm{mech}}$. Note that enthalpy or other chemical contributions arising in polymeric systems can be included here.

The set of species $I^{\mathrm{sel}}$ which are present in $\Omega^{\mathrm{sel}}$ decomposes into the mobile species $I^{\mathrm{pass}}$ which are allowed to pass the filter, mobile species $I^{\mathrm{conf}}$ that diffuse but are confined in the selectivity filter, and the immobile $I^{\mathrm{scaf}}$ species which actually form the scaffold structure of the filter and thus the lattice sites on which the mobile species may diffuse [29].

We consider thus a mixture of particles on a lattice which is formed by species $A_{\alpha }$, $\alpha \in I^{\mathrm{scaf}}$ and on which the particles $A_{\alpha },\alpha \in I^{\mathrm{pass}}\cup I^{\mathrm{conf}}$ may mix. Let $N_{\alpha },\alpha \in I^{\mathrm{sel}}$ denote particle numbers and $N_{\ell }=\sum_{\alpha \in I^{\mathrm{scaf}}}\omega_{\alpha }^{\mathrm{scaf}}N_{\alpha }$ the number of lattice sites, whereby $\omega_{\alpha }^{\mathrm{scaf}}$ is the number of lattice sites each particle $A_{\alpha },\alpha \in I^{\mathrm{scaf}}$ provides. Further, we assume that each constituent $A_{\alpha },\alpha \in I^{\mathrm{pass}}$ requires $\omega_{\alpha }^{\mathrm{pass}}$ sites on the lattice and each constituent $A_{\alpha },\alpha \in I^{\mathrm{conf}}$ requires $\omega_{\alpha }^{\mathrm{conf}}$ sites on the lattice, whereby the number of vacancies (free lattice sites) is

(A7) $$\begin{array}{cc} N_{V} & =N_{\ell }-\sum_{\alpha \in I^{\mathrm{pass}}}\omega_{\alpha }^{\mathrm{pass}}N_{\alpha }-\sum_{\alpha \in I^{\mathrm{conf}}}\omega_{\alpha }^{\mathrm{conf}}N_{\alpha }. \end{array}$$

The number of entropically exchangeable particles $\tilde{N}$ is hence

(A8) $$\begin{array}{c} \tilde{N}=N_{V}+\sum_{\alpha \in I^{\mathrm{pass}}}N_{\alpha }+\sum_{\alpha \in I^{\mathrm{conf}}}N_{\alpha }. \end{array}$$

This leads to the number of possible configurations (note that this is the multi-nominal coefficient.)

(A9) $$\begin{array}{c} W=\left(\begin{array}{c} \tilde{N}\\ N_{1},\ldots ,N_{N^{\mathrm{pass}}},N_{1},\ldots ,N_{N^{\mathrm{conf}}},N_{V} \end{array}\right) \end{array}$$

where we assumed $I^{\mathrm{pass}}=\left\{1,2,\ldots ,N^{\mathrm{pass}}\right\}$ and $I^{\mathrm{conf}}=\left\{1,2,\ldots ,N^{\mathrm{conf}}\right\}$ for the sake of this derivation. The entropy of mixing is then

(A10) $$\begin{array}{c} S=k_{B}\left(\begin{array}{c} \tilde{N}\\ N_{1},\ldots ,N_{N^{\mathrm{pass}}},N_{1},\ldots ,N_{N^{\mathrm{conf}}},N_{V} \end{array}\right), \end{array}$$

which leads in the Stirling approximation to the configuration entropy

(A11) $$\begin{array}{c} S=-k_{B}\;\left(\sum_{\alpha \in I^{\mathrm{pass}}}N_{\alpha }\;\ln\left(\frac{N_{\alpha }}{\tilde{N}}\right)+\sum_{\alpha \in I^{\mathrm{conf}}}N_{\alpha }\;\ln\left(\frac{N_{\alpha }}{\tilde{N}}\right)+N_{V}\;\ln\left(\frac{N_{V}}{\tilde{N}}\right)\right). \end{array}$$

Transition to particle molar densities $n_{\alpha }=N_{\alpha }/\left(VN_{A}\right),\;\alpha =I^{\mathrm{sel}}\cup V$, where *V* denotes the volume of the *mental* box, leads to a configurational entropy contribution of the free energy as

(A12) $$\begin{array}{c} \rho \psi^{\mathrm{sel},\mathrm{mix}}=\sum_{\alpha \in I^{\mathrm{pass}}}n_{\alpha }\;RT\;\ln\left(\frac{n_{\alpha }}{\tilde{n}}\right)+\sum_{\alpha \in I^{\mathrm{conf}}}n_{\alpha }\;RT\;\ln\left(\frac{n_{\alpha }}{\tilde{n}}\right)+n_{V}\;RT\;\ln\left(\frac{n_{V}}{\tilde{n}}\right). \end{array}$$

with $\tilde{n}$, $n_{\ell }$ and $n_{V}$ given by Equations (17), (18) and (19), respectively.

Note that for a single diffusive species $y=n_{\alpha }/\tilde{n}$ requiring one lattice site, one obtains the simple lattice entropy of mixing

(A13) $$\begin{array}{c} \rho \psi^{\mathrm{sel},\mathrm{mix}}=n_{\ell }RT\;\left(y\ln\left(y\right)+\left(1-y\right)\ln\left(1-y\right)\right). \end{array}$$

The mechanical contribution is considered as

(A14) $$\begin{array}{c} \rho \psi^{\mathrm{sel},\mathrm{mech}}=\left(K^{\mathrm{sel}}-p^{R}\right)\left(1-H^{\mathrm{sel}}\right)+K^{\mathrm{sel}}H^{\mathrm{sel}}\ln\left(H^{\mathrm{sel}}\right), \end{array}$$

with $H^{\mathrm{sel}}=\sum_{\alpha \in I^{\mathrm{scaf}}}v_{\alpha }^{\mathrm{sel}}n_{\alpha }$, whereby only the immobile species contribute to the mechanical energy. The reference contribution is similar to the electrolyte considered as

(A15) $$\begin{array}{c} \rho \psi^{\mathrm{ref}}=\sum_{\alpha \in I^{\mathrm{sel}}}g_{\alpha }^{\mathrm{sel}}\;n_{\alpha }. \end{array}$$

In the incompressible limit $K^{\mathrm{sel}}\to \infty$, we obtain the chemical potential functions given by Equations (16a), (16b) and (16c).

Regarding the *backbone charge* of the *scaffold* structure, some cases have to be considered:

- backbone is uncharged, whereby
  (A16) $$\begin{array}{c} q^{\mathrm{sel}}=F\sum_{\alpha \in I^{\mathrm{pass}}\cup I^{\mathrm{conf}}}z_{\alpha }^{\mathrm{sel}}n_{\alpha } \end{array}$$
- backbone is fully charged, whereby
  (A17) $$\begin{array}{c} q^{\mathrm{sel}}=F\sum_{\alpha \in I^{\mathrm{pass}}\cup I^{\mathrm{conf}}}z_{\alpha }^{\mathrm{sel}}n_{\alpha }+Fz_{n_{\ell }}n_{\ell } \end{array}$$
  and $z_{n_{\ell }}<0$. Note that $q^{\mathrm{sel}}$ has then a constant backbone volume charge, similar like a solid electrolyte. This has to satisfy then (integrate poisson equation, initial state is that there are no $A_{\alpha },\;\alpha \in I^{\mathrm{pass}}\cup I^{\mathrm{conf}}$ present within $\Omega^{\mathrm{SF}}$)
  (A18) $$\begin{array}{c} Fz_{n_{\ell }}n_{\ell }\;\mathrm{vol}\left(\Omega^{\mathrm{sel}}\right)\overset{!}{=}\;{\underset{s}{q}}^{\mathrm{sel},\mathrm{lip}}\;\mathrm{area}\left(S^{\mathrm{sel},\mathrm{lip}}\right), \end{array}$$
  where $\;{\underset{s}{q}}^{\mathrm{sel},\mathrm{lip}}$ is the surface charge of the channel protein within the selectivity filter.
- an intermediate state, whereby
  (A19) $$\begin{array}{c} Fz_{0}^{\mathrm{sel}}n_{\ell }\;\mathrm{vol}\left(\Omega^{\mathrm{sel}}\right)\overset{!}{=}\zeta \;\;{\underset{s}{q}}^{\mathrm{sel},\mathrm{lip}}\mathrm{area}\left(S^{\mathrm{sel},\mathrm{lip}}\right), \end{array}$$
  with some scaling parameter ζ.

Note that electroneutrality must also be ensured in the selectivity filter when charged species are present and backbone and surface charges are considered.

## Appendix B. Parameter Values

For all constants and parameter values that were not explicitly mentioned in the text we used the values given in Table A1.

**Table A1:** Parameter values used to simulate an calcium-selective ion channel.

Symbol	Meaning	Value	Unit
*T*	temperature	298.15	K
$e_{0}$	elementary charge	1.602 $\times \;10^{-19}$	C
$k_{B}$	Boltzmann constant	1.380 $\times \;10^{-23}$	J $K^{-1}$
$N_{A}$	Avogadro constant	6.022 $\times \;10^{23}$	${\mathrm{mol}}^{-1}$
$F=e_{0}N_{A}$	Faraday constant	9.648 $\times \;10^{4}$	C ${\mathrm{mol}}^{-1}$
$R=k_{B}N_{A}$	Gas constant	8.314	J ${\mathrm{mol}}^{-1}$ $K^{-1}$
$\varepsilon_{0}$	vacuum permittivity	8.854 $\times \;10^{-12}$	F $m^{-1}$
$\varepsilon_{r}^{j}=1+\chi^{j}$	relative permittivity	78.49	-
$D_{{\mathrm{Ca}}^{2+}}^{j}$, $D_{{\mathrm{Na}}^{+}}^{j}$, $D_{{\mathrm{Cl}}^{-}}^{j}$	diffusion coefficients	$\left[0.79,1.334,2.032\right]\times 10^{-9}$	$m^{2}$ $s^{-1}$
$D_{{\mathrm{Ca}}^{2+}}^{\mathrm{sel}}$, $D_{{\mathrm{Na}}^{+}}^{\mathrm{sel}}$ in $\Omega^{\mathrm{sel}}$	diffusion coefficients	$\left[0.198,2.0\right]\times 10^{-12}$	$m^{2}$ $s^{-1}$
$z_{{\mathrm{Ca}}^{2+}}$, $z_{{\mathrm{Na}}^{+}}$, $z_{{\mathrm{Cl}}^{-}}$	charge numbers	2, 1, −1	-
$M_{H_{2}O}$	molar weight water	18.0	g ${\mathrm{mol}}^{-1}$
$M_{{\mathrm{Ca}}^{2+}}$, $M_{{\mathrm{Na}}^{+}}$, $M_{{\mathrm{Cl}}^{-}}$	molar weights	40.1, 23.0, 35.5	g ${\mathrm{mol}}^{-1}$
$v_{H_{2}O}$	molar volume water	55.4	$m^{3}$ ${\mathrm{mol}}^{-1}$
$v_{{\mathrm{Ca}}^{2+}}$, $v_{{\mathrm{Na}}^{+}}$, $v_{{\mathrm{Cl}}^{-}}$	molar volumes	[26.20, 23.78, 17.39] $\times \;10^{-6}$	$m^{3}$ ${\mathrm{mol}}^{-1}$
$\;{\underset{s}{q}}^{\mathrm{sel}}$	charge channel wall	$-2e_{0}$	${\mathrm{nm}}^{-2}$
[${\mathrm{CaCl}}_{2}{]}^{\mathrm{in}}$, [${\mathrm{CaCl}}_{2}{]}^{\mathrm{out}}$	bulk concentrations	0, varying	mM
[${\mathrm{NaCl}]}^{\mathrm{in}}$, [NaCl${]}^{\mathrm{out}}$	bulk concentrations	32, 32	mM
$\varphi^{\mathrm{in}}$, $\varphi^{\mathrm{out}}$	bulk potential	−20, 0	mV
$\kappa_{{\mathrm{Ca}}^{2+}}$, $\kappa_{{\mathrm{Na}}^{+}}$, $\kappa_{{\mathrm{Cl}}^{-}}$	solvation numbers	30, 60, 30	-
*r*	channel radius	4.5	Å
*l*	length of $\Omega^{\mathrm{sel}}$	14	Å
$l_{\mathrm{charge}}$	length of $S^{\mathrm{charge}}$	8	Å
$n_{\ell }$	lattice sites	2	M
$n_{O}$	oxygen ions in $\Omega^{\mathrm{sel}}$	0	M
