# Supplementary material for: A Model Framework for Ion Channels with Selectivity Filters Based on Non-Equilibrium Thermodynamics
Source: Entropy (Basel). 2025 Sep 20;27(9):981. doi: 10.3390/e27090981 (PMC12469145; doi:10.3390/e27090981)
Supplement: Supplementary file 1 [file entropy-27-00981-s001.zip › data/ReadMe_Data_Figure_4.rtf]

The data for Figure 4 can be read and loaded with the Julia package DrWatson.jl (doi: 10.21105/joss.02673) as followsdatadict = wload("myfile.jld2")datagrid = datadict["grid"]solution = datadict["solution"]datagrid contains the 2d gridsolution contains the 2d solutions and is of the following form (nQ,N,Nx), where the column nQ contains all solutions for different surface charges, N is the number of variables and Nx is the number of grid pointsnQ = 1: q=0nQ = end: q=-2N = 1: solution for sodiumN = 2: solution for calciumN = 3: solution for chlorideN = 4: solution for phiN = 5: solution for pressure   
